# Orienting selective attention within long-term contextual memories

**DOI:** 10.1038/s44271-026-00505-9

**Published:** 2026-07-16

**Authors:** Melinda Sabo, William Narhi-Martinez, Nahid Zokaei, Nora M. Roüast, Anna C. Nobre

**Affiliations:** 1 Department of Psychology and Wu Tsai Institute, Yale University, New Haven, CT USA; 2 Departments of Experimental Psychology and Psychiatry, Oxford Centre for Human Brain Activity, University of Oxford, Oxford, UK

**Keywords:** Human behaviour, Long-term memory, Human behaviour

## Abstract

Selective attention is central to long-term memory retrieval, yet how attention selects and prioritizes relevant memory contents remains underexplored. Well-controlled experimental designs aimed at isolating and systematically manipulating attentional orienting in long-term memory would greatly advance this cause. The current study addresses this gap. Building on the precue and retrocue designs from working-memory research, we introduce an experimental framework to investigate how selective attention operates within contextual long-term memory. Across three pre-registered experiments (*n* = 235), participants learned associations between scenes and the identities and locations of two objects. In a subsequent Selective Retrieval task, participants viewed the studied scenes and later identified scene-associated objects from an array. Spatial cues directed internal attention to the location of the object to be probed, or neutral cues offered no spatial information. Informative cues yielded significant retrieval benefits and costs relative to neutral cues. Benefits emerged regardless of whether cues appeared before or after scene presentation and were fully expressed even with very short retrieval intervals. The approach offers a flexible method for isolating and manipulating attention functions in long-term memory, enabling future investigation of the psychological and neural mechanisms governing selective memory access.

## Abstract (web summary)

We show that valid spatial cues directing internal attention during long-term memory retrieval boost recall, while invalid cues incur costs. Our work offers a flexible experimental framework to study selective attention in long-term memory.

## Introduction

The strong interconnection between memory and attention has been recognized since the dawn of experimental psychology^1–4^. Today, many proposals exist regarding the precise definitions and interrelations between memory and attention^5–13^.

Recent treatments have suggested that memory and attention work together to guide adaptive behavior^14^. In our framework, memories of different types and lifetimes are proposed to furnish experience-related traces. Selective attention is the set of functions that utilizes these memory traces to anticipate, select, prioritize, and prepare contents to shape perception, cognition, and action^15^. In this framework, attention can work on contents originating in the sensory stream (external) or on pre-existing representations untethered to sensory signals, such as memory contents (internal).

Attention functions are most often investigated in the context of processing external sensory stimuli. For example, top-down signals from working memory representations of task goals modulate several stages of sensory and motor processing to enhance task performance^16,17^. Increasingly, however, the workings of internal attention directed toward memory contents are also gaining scientific interest^14,18,19^. The ability to direct selective attention within working memory based on locations, features, timings, or associated actions is well established by now^15^. Investigations are turning toward elucidating the various neural mechanisms through which the brain selects, prioritizes, and prepares working-memory contents for the impinging future depending on memory attributes and task demands^20–23^.

In contrast, our knowledge of internal attention in long-term memory is more limited. “That” attention plays a core role in long-term memory is hardly questioned. Some even propose that selective retrieval is equivalent to internal attention in long-term memory^9,18^. We routinely retrieve selective aspects of individual memory experiences from boundless competing traces stored in mind. Remembering the friend with whom you dined at your first conference can feel natural but requires an attention tour de force.

The central role that selective attention plays in successful long-term retrieval is acknowledged across many strands of evidence^24–28^. For example, work investigating divided attention during long-term memory retrieval suggested that competing attentional demands from concurrent tasks or perceptual distractors impair retrieval performance^29–34^. Thus, divided-attention manipulations show that retrieval operations involve attentional resources. Other lines of research indicate that memory contents may compete and influence one another^35–39^. When two overlapping associations are learned (A-B, A-C), and one is selectively retrieved over the other one, memory performance and neural traces for the non-retrieved pair are diminished relative to a baseline condition^9,36,40–42^. Finally, various experimental protocols have highlighted attention functions operating during memory formation and retrieval. For example, work focusing on the phenomenon of suppression-induced forgetting suggests that individuals can suppress memory associations voluntarily based on instructive cues^43,44^.

Neural measures have also supported the engagement of attention-related regions during memory retrieval. Successfully retrieving a previously weakened, selected-against memory, engages the prefrontal and anterior cingulate cortices^45^. Parietal regions have also been found to be activated in retrieval tasks. Previous studies have suggested that it is possible to decode memory content from dorsal frontal and parietal activation patterns, with the signal strength modulated by the retrieval goal^46,47^. According to one model, the parietal activations during memory retrieval reflect top-down and bottom-up attention functions equivalent to those found in external attention (Attention to Memory model^24^). However, the extent of overlap between parietal areas during memory retrieval and external attention continues to be debated^27,48^. Human neurophysiology studies have also revealed EEG markers of attention during long-term memory retrieval. Modulations of event-related potentials have been linked to retrieval orientation, which entails biasing memory search in service of goals^49–53^. Other work has suggested that upon presentation of memory retrieval cues, EEG markers of attention are enhanced for spatial associations that were signaled as relevant compared to the irrelevant ones^54–57^.

Thus, various research strands converge on demonstrating ***that*** selective attention plays a central role in long-term memory retrieval. The critical missing tool is a reliable and flexible experimental framework that would allow the isolation and manipulation of attentional processes during long-term memory retrieval. Such an experimental tool would additionally contribute to the investigation of ***how*** attention operates during retrieval.

As a way forward, we introduce an experimental framework, which will allow the investigation of psychological and neural properties of selective attention in long-term memory. The proposed experimental framework builds on the successful field of internal selective attention within working memory^14,23,58^, where the “retrocue” has provided an effective tool for manipulating attentional orienting. Retrocues are informative cues typically presented during the working-memory maintenance period, which retroactively predict which specific content will be relevant for the subsequent task. Retrocueing studies have revealed extraordinary flexibility in orienting and reorienting attention in working memory based on spatial location, objects, features, and timings of relevant content. These studies have also opened the doors for a granular exploration of the multiple neural mechanisms involved^15,20^.

Adapting retrocue manipulations to investigate internal attention in long-term memory requires the formation of controlled memory representations with competing contents. To achieve this, we built on previous experimental designs that investigated long-term memory guidance of external attention^59–64^ and item competition in long-term memory^37–39,42^. In the current study, individuals learn to identify and locate two unique objects embedded within scenes. After these controlled memories have been formed, we test the ability of informative spatial cues to orient attention to a specific object within the scene.

The approach isolates the effects of attention, which is important but not trivial. Although attention functions are central, or even intrinsic to retrieval, they operate alongside other functions. In addition to selective attention, retrieval often involves motivation, strategic planning, pattern completion, evidence accumulation, decision making, evaluating outcomes, affective consequences, and different degrees of awareness.

Thus, our aim is to extend the experimental toolkit of internal-attention retrocueing, developed for working-memory contents, to contents reactivated from long-term memory. Exploring internal attention within long-term memory should open doors to important and longstanding questions. For example, if cueing significantly enhances selective long-term-memory retrieval, future studies will be able to test directly the role of working memory in this process. Three scenarios are possible: (1) Selective long-term-memory retrieval requires the initial reinstatement into working memory with internal attention operating in the same way as it does on working memory contents encoded from the sensory stream. (2) Selective long-term-memory retrieval requires the initial reinstatement into a working memory buffer. However, contents reinstated from long-term memory may differ in their qualities or properties compared to items freshly encoded into working memory from the sensory stream Therefore, the consequences of internal attention may also differ systematically. (3) Finally, internal attention may operate directly on long-term memory representations independently of working-memory reinstatement, modulating retrieval in distinct ways.

To establish the efficacy of the experimental approach, we conducted three experiments. Throughout these experiments, we aimed to test whether spatially informative cues could guide the selection of relevant long-term memory content and thereby improve retrieval performance. Participants learned associations between unique scenes and the identities and locations of two objects. In the subsequent Selective Retrieval task, participants briefly viewed each studied scene. At the end of the trial, they chose which of two probe items was associated with the scene. Spatial cues provided information about what item would be probed either after (Experiment 1a and 1b) or before scene presentation (Experiment 2). In contrast, neutral cues offered no such information. The first experiment (Experiment 1a) probed recognition of studied objects compared to new, unstudied items. Participants could therefore rely on familiarity or associative recollection. The subsequent experiments tapped into associative recollection, requiring participants to retrieve the object specifically associated with the probed scene.

We predicted that informative cues would significantly impact long-term memory retrieval performance. As stated in our preregistration, we hypothesized that performance would be significantly better for spatial cues correctly indicating the to-be-probed item (valid cue) compared to cues pointing to the wrong object (invalid cue). Neutral cues were included to dissect the putative benefits (higher performance in the valid vs. neutral condition) and costs (higher performance in the neutral vs. invalid condition) of internal attention.

In Experiment 2, the timing of the cues was also manipulated to test whether attentional benefits could emerge when the retrieval time is limited. The retrieval-interval manipulation provided a first foray into the fascinating and longstanding question about the different stages at which selection operates within retrieval.

In principle, the selection and prioritization of memory contents might operate at three different stages. (1) Attention functions may start modulating long-term memory contents very early on, before the representations are transferred to working memory. This is plausible, given the existence of functional anatomical connections between long-term memory control regions (e.g., in medial temporal areas) and visual regions required for retrieving the sensory qualities of the associated contents. In this scenario, selection and prioritization of long-term-memory contents may occur before they reach an active state in working memory, recruiting prefrontal and parietal areas. (2) Alternatively, long-term memory retrieval may necessarily involve reinstatement and mediation via working memory. Selection and prioritization may occur only after the long-term memory contents have been transferred to working memory. (3) A final possibility is that attention functions do not impact the retrieval process itself, but instead operate later, at the response stage. In this case, attentional processes may help guide the decision of which retrieved content is relevant or correct. These three possibilities are not mutually exclusive, and there could be hybrid or other variations.

By using long (800 ms) or short (100 ms) retrieval intervals in Experiment 2, we manipulated the time available to retrieve and prioritize the scene-associated objects before the probe array appeared. We reasoned that the long interval was likely to afford the required time to reinstate contents into working memory before the probe, whereas the short interval was likely to compromise full reinstatement. We hypothesized that if attention functions can operate very early in the retrieval process (scenario 1), cueing effects should be comparable irrespective of the retrieval interval. In this case, no interaction between cueing condition and retrieval interval would be expected. Alternatively, if selective attention only acts after the long-term memory content is in working memory (scenario 2), its benefits should be intact with a long retrieval interval but would be compromised with a short retrieval interval. This would occur because, in the short interval condition, reinstatement would not be complete when the probe appears. However, we cannot exclude the possibility that the processes of memory reinstatement and attentional prioritization continue to unfold after the probe presentation. Finally, if attention within long-term memory acts only at the response stage (scenario 3), this would yield an inconclusive pattern of results. We therefore treat the Experiment 2 results as a first behavioral foray into the time course of attentional modulation during long-term memory retrieval, not as a definitive test of working-memory mediation.

To foreshadow our results, all experiments revealed reliable benefits of orienting selective attention in long-term memory. Furthermore, the benefits of attention were similar for both long and short retrieval intervals. The experimental approach opens the door to exciting questions regarding how attention guides selective retrieval in long-term memory and for probing the underlying psychological and neural mechanisms.

## Methods

The experimental procedure was approved by the Yale University Institutional Review Board. Both Experiments 1 and 2 were preregistered: https://osf.io/e4vyq (Experiments 1a and 1b, preregistered on September 8^th^, 2025) and https://osf.io/drf29 (Experiment 2, preregistered on October 20^th^, 2025). All data and analyses scripts are available: https://osf.io/k9xu7.

### Participants

Demographic information for all three experiments is reported in Table 1. As specified in the preregistration, the sample size was estimated using a power analysis conducted in G*Power (paired samples, two-tailed, α = 0.05, power = 0.80). This analysis was based on pilot data from a separate experiment measuring valid vs. invalid retrocue effects in long-term memory. The analysis indicated that 37 participants would be required. Because the planned experiments introduced several adjustments and tested more subtle effects (i.e., validity benefits and costs specifically) under conditions of likely greater performance variability than the pilot, we adopted a more conservative sampling strategy. We initially recruited 60 participants per experiment and calculated Bayes factors (BF₁₀) to test for conclusive evidence for attention benefits and costs during the Selective Retrieval task. We compared accuracy (Experiment 1) and response times (Experiments 1 and 2) between the valid and neutral conditions. If necessary, additional participants were recruited, in increments of five, until the evidence became conclusive for the alternative (BF_10_ > 5.0) or the null (BF_10_ < 0.20) hypothesis. The procedure followed other behavioral studies conducted in the memory domain^65^ and was in line with our preregistration. The final sample sizes were 60 participants in Experiment 1a, 90 in Experiment 1b, and 85 in Experiment 2.

**Table 1:** Demographic information

Experiment	*N*	Gender (F / M / O)	Mean age	SD age	Age range
1a	60	20 / 39 / 1	29.73	5.99	18–39
1b	90	25 / 65 / 0	30.51	5.44	19–40
2	85	31 / 54 / 0	29.33	5.65	20–40

Gender was reported by participants as female (F), male (M), or other (O).

Participants were recruited through the Prolific platform (www.prolific.co). The researcher involved in the data collection had access to each participant’s race information, however, this has not been considered and analyzed. No SES data were collected. Inclusion criteria comprised being between 18 and 40 years old, speaking English fluently, completion of at least 10 prior experiments, and having an approval rate of minimum 95%. All participants reported normal or corrected vision, no difficulties with color vision, and no mental health diagnosis. Before completing the study, they provided informed consent digitally through the Qualtrics platform. Participants were compensated for their time at an hourly rate of $12.

In line with the preregistration, data were only included if participants completed the entire experiment, achieved at least 90% accuracy in the final block of the Learning task (Experiment 1), and had a minimum of ten trials in each condition of the Selective Retrieval task. Participants also needed to exceed 60% accuracy in the neutral cue condition of the Selective Retrieval task and indicate at the end of the study in the follow-up questionnaire that their data should be used in the study. Finally, if a participant did not reach at least 60% performance in the Selective Retrieval task of the first experimental session, they were not re-invited to the second session.

### Stimuli

The experiments used randomly selected color photographs (1080×720 pixels) of indoor and outdoor scenes obtained from https://stock.adobe.com/ and object stimuli from the experimental set compiled by Brady and colleagues^66^. Experiments 1a and 1b used 80 scenes, and Experiment 2 used 96 scenes. To ensure compatibility with online testing, all images were downsized to 250 kB or less. Their vibrance was also reduced to better match the vibrance of the object stimuli. Experiment 1a included 340 objects (160 for the Learning task and 180 for the Selective Retrieval task), Experiment 1b included 160 objects, and Experiment 2 included 196 objects. Objects were randomly selected from categories such as toys, utensils, food, and other everyday items. Images were processed to remove their background, and a 7-pixel white border was added. All scripts used for stimulus processing are available (https://osf.io/k9xu7). A further 8 scenes and 20 objects, sourced in the same way but different from those used in the main tasks, were used for the practice trials.

### Procedure

Within each experiment, participants completed two identical experimental sessions, scheduled no more than seven days apart. Each session used entirely different sets of scenes and objects, with no overlap between days. Splitting the procedure across two days allowed us to increase the total number of trials without making a single session excessively long. In each session, participants learned 40 or 48 scene-object associations. The scene and object stimuli used in the two experimental sessions were different, so that there could be no memory-related contamination.

All experiments included two tasks (described below), separated by a five-minute Tetris game. Before the experiment, participants filled out a demographic questionnaire, read task instructions, and completed a brief training phase to become familiar with the procedure. At the end of the study, they completed a short follow-up questionnaire. The experiments were programmed in PsychoPy (version v. 2024.2.4)^67^ and administered online via Pavlovia (pavlovia.org).

#### Learning task

**Fig. 1:**
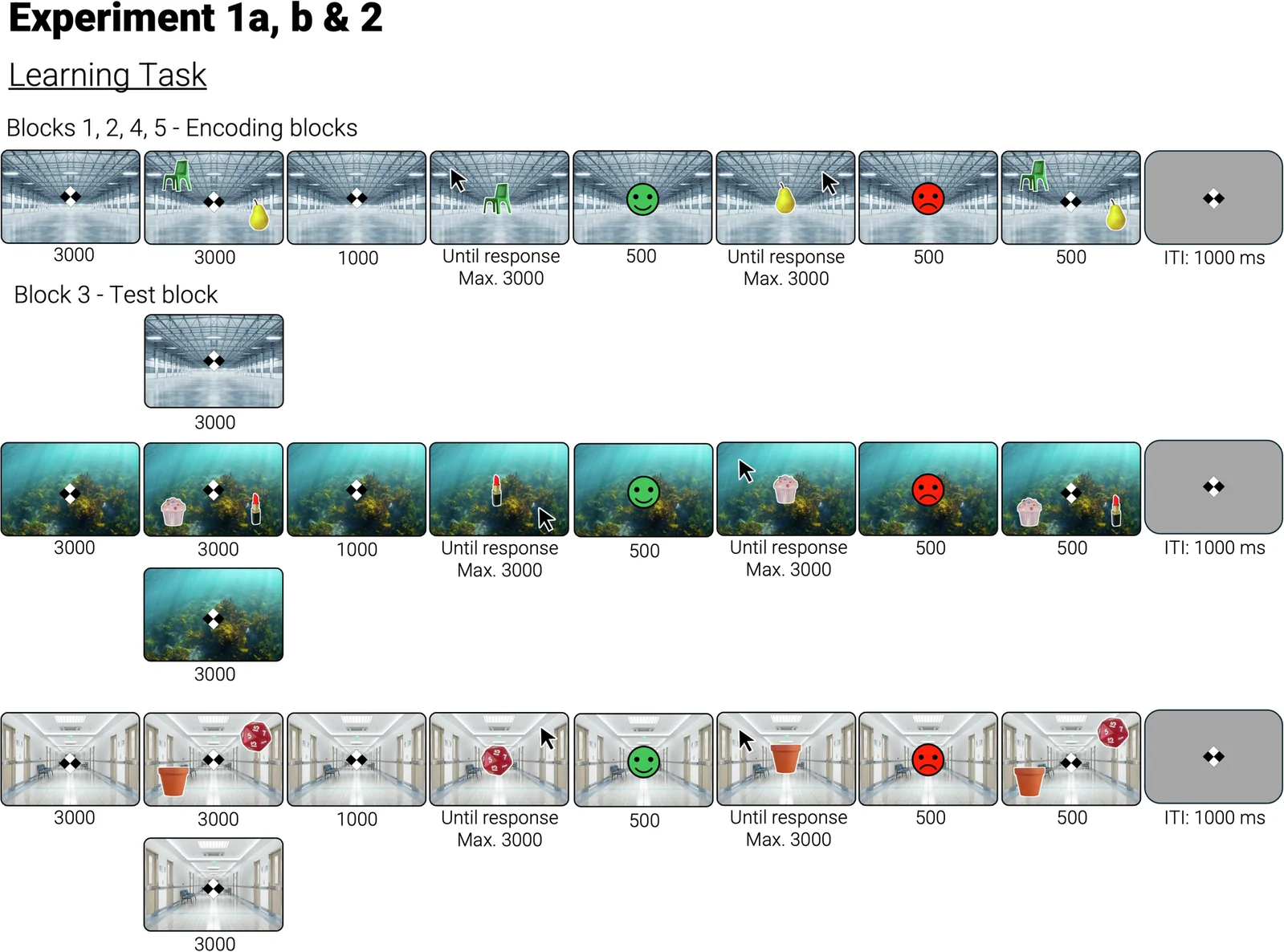
Learning task design – Experiment 1a, b and 2. Participants learned the identity and location of two objects embedded within scenes (experiment 1: 80 scenes, experiment 2: 96 scenes). They completed four encoding blocks and one test block. Each encoding trial began with a scene containing two embedded objects. After viewing the scene, participants were asked to indicate the location of each previously encountered object using the mouse. Each response was followed by feedback. At the end of the trial, participants were again shown the scene together with the two embedded objects. In the test block, the same procedure was used but the scenes were presented without embedded objects, requiring retrieval of the location from long-term memory.

The Learning task was equivalent across all three experiments (Fig. 1). Participants learned to associate the identity and location of two unique objects with a scene. Within each scene, one object was on the left and the other on the right side. Objects could occupy one of four positions on each side. Object locations were: upper left (PsychoPy units: −0.325, 0.188 and −0.188, 0.325), lower left (PsychoPy units: −0.325, −0.188 and −0.188, −0.325), upper right (PsychoPy units: 0.325, 0.188 and 0.188, 0.325), and lower right (PsychoPy units: 0.325, −0.188 and 0.188, −0.325). The eight locations were counterbalanced and equally distributed across different objects and trials. The scene-object associations were held constant across all learning blocks for a given participant, but they were randomly assigned across participants.

The Learning task consisted of five blocks. Blocks 1, 2, 4, and 5 were encoding blocks, and Block 3 served as a test block. At the beginning of each Learning trial in encoding blocks, participants viewed a scene (size: 1.75 × 1 PsychoPy units) for 3000 ms, followed by the appearance of two objects (size: 0.20 × 0.20 PsychoPy units) overlaid on the scene for an additional 3000 ms. They were instructed to remember both the identity and the location of the objects associated with each scene. After a 1000-ms delay, the first probe array appeared. Participants were shown one of the scene-associated objects and asked to place it at its remembered location using the mouse. If the reported location fell within the correct quadrant, a green smiley face (size: 0.75 × 1 PsychoPy units) was displayed for 500 ms. Otherwise, a red sad face indicated an incorrect response. If responses were not provided within 3000 ms, participants were shown a red smiley face. Trials with incomplete responses were removed from the analyses. Next, a second probe array appeared. Participants were shown the other scene-associated object and were asked to indicate its location. Feedback was provided in the same way as for the first probe. At the end of each trial, the scene and the two objects were presented again for 500 ms. The next trial began after a 1000-ms inter-trial interval, during which only fixation was present.

The test block checked how well participants learnt the scene-object associations, while also aimed to motivate them to improve encoding. Trials were identical to those in the encoding blocks, except that the objects did not appear superimposed on the scene after the initial 3000-ms scene presentation. Instead, participants viewed the scene for an additional 3000 ms (Fig. 1) and were required to recall the object locations from long-term memory. All scene-object associations were tested in this block.

After completing the Learning task, participants played Tetris for five minutes. In Tetris, players rotate and position falling blocks to complete horizontal lines, which then disappear. This distractor phase was intended to prevent rehearsal of the learned visual associations by engaging participants in an intensive and active visual-spatial task^68^. After five minutes had elapsed, participants proceeded to the Selective Retrieval task.

#### Selective retrieval task

Across the three experiments, the Selective Retrieval task examined whether internal spatial attention could influence object retrieval. Participants first viewed a previously studied scene. At the end of the trial, they were asked to retrieve the object previously associated with the scene. The probe array contained two objects, and participants were required to make a two-alternative forced-choice (2AFC) response. The 2AFC method was chosen for its sensitivity to the presence of memory traces, the reduced impact of changes in response criteria, and its robustness for distinguishing target vs. non-target memory distributions. Between the scene and the probe array, participants were presented informative or neutral retrocues (Experiments 1a and 1b). In Experiment 2, the informative or neutral cues were shown before scene presentation (precues). Details of stimulus timings, cue validity, and probe types differed across experiments to address specific, interrelated research questions.

##### Experiment 1a

**Fig. 2:**
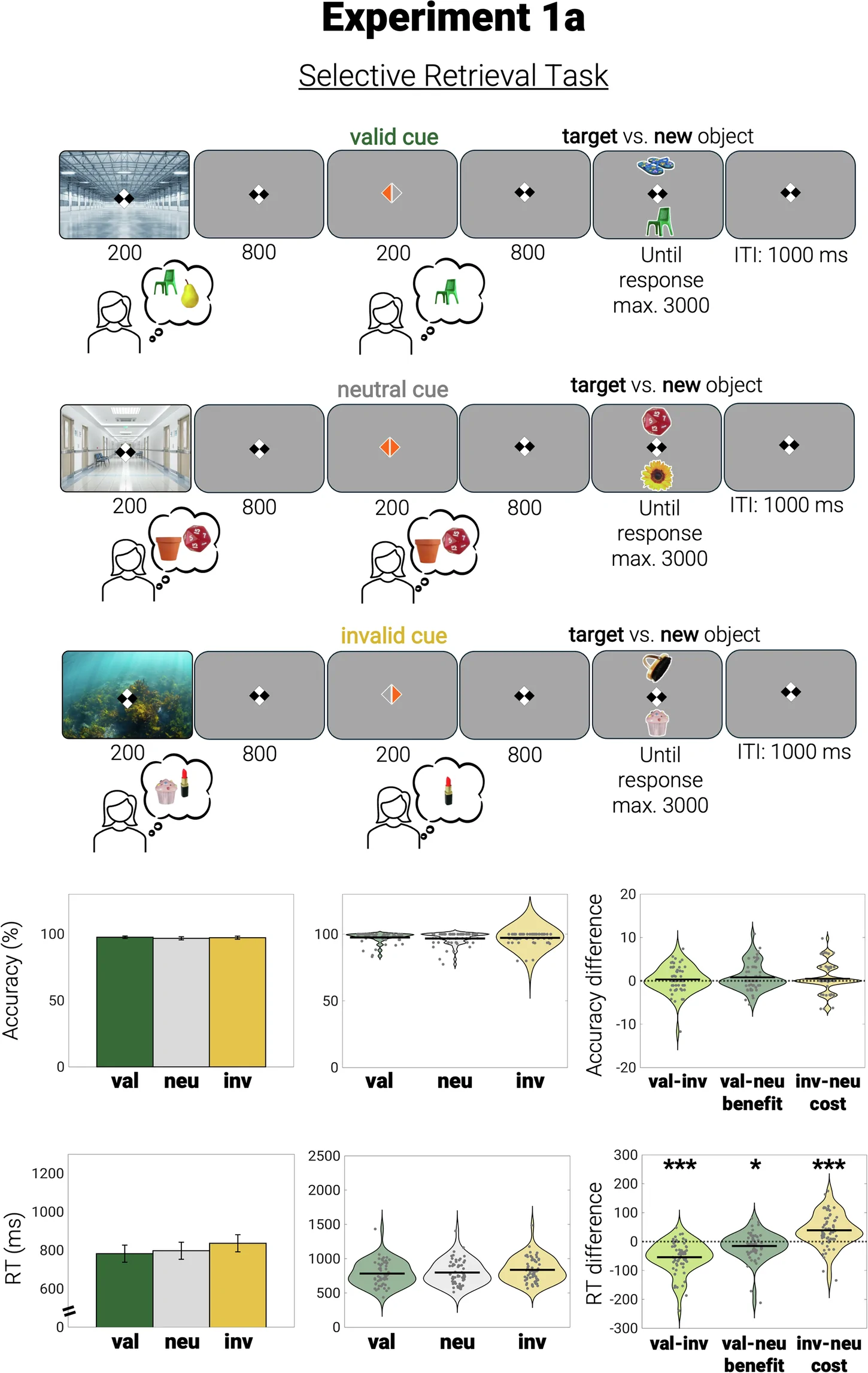
Selective Retrieval task design and results – Experiment 1a (*N* = 60). Top panel: experimental design. In Experiment 1a, the Selective Retrieval task used 75% valid spatial retrocues. At the beginning of the trial, participants were shown a previously studied scene. After an 800 ms delay period, they were briefly shown a valid, invalid or neutral cue. At the end of the trial, a probe array appeared on screen and participants were required to select the object associated with the scene (2AFC decision). Since the nontarget object was a previously unseen object, the test is equivalent to an old-new judgment. The bottom panel illustrates accuracy and response times results. While accuracy was at ceiling (above 95%), and showed no significant effects, response times results revealed both attentional benefits, as well as attentional costs. Both the bar plots and the violin plots (center) depict accuracy and response times contrasts between conditions, as indicated by the labels. Error bars in the bar plots represent the standard error of the mean. The right-side violin plots illustrate the three condition differences. The black solid line represents the condition means (middle panel) or the mean condition differences (right panel). Statistical significance is indicated with: *p < 0.05, **p < 0.01, ***p < 0.001.

Experiment 1a was designed to measure the putative benefits and costs of spatial retrocues in the context of an old/new recognition judgment. A schematic of the Selective Retrieval task in Experiment 1a is presented in Fig. 2. Participants first viewed a previously studied scene from the Learning task for 200 ms. This was followed by a delay of 800 ms, before the presentation of a retrocue (200-ms duration; size: 0.1 × 0.1). Retrocues were either informative or neutral. Informative retrocues occurred on the majority of trials and predicted the side on which the to-be-probed object had appeared in the scene. The left or right side of the cue was highlighted in orange, pointing to the correct location on 75% of trials. On the remaining 25%, the object from the uncued side appeared in the probe array. Neutral cues were highlighted in orange on both sides and provided no information about which object was likely to be probed. Participants were explicitly told that the cue is invalid in a proportion of trials.

In Experiment 1a, the probe array contained one previously studied object and one previously unseen item. Objects in the probe array were arranged above (PsychoPy locations: 0, 0.25) and below (0, −0.25) fixation. The locations of the target and non-target objects were counterbalanced in the probe array. Participants responded using the upper or lower arrow key on the keyboard. They were instructed to respond as quickly and as accurately as possible. The response window was 3000 ms. No feedback was provided during the Selective Retrieval task.

In each experimental session, participants completed two blocks of the Selective Retrieval task. Each block tested retrieval for each of the previously studied scenes in a randomized order. Across the two experimental sessions, the retrieval of associated objects was tested twice for each of the 80 scenes (160 trials across the two experimental sessions). Overall, participants completed 96 trials with valid informative retrocues, 32 trials with invalid informative retrocues, and 32 trials with neutral retrocues. The assignment of each scene to a condition and the selection of the target object for each block were determined randomly.

##### Experiment 1b

**Fig. 3:**
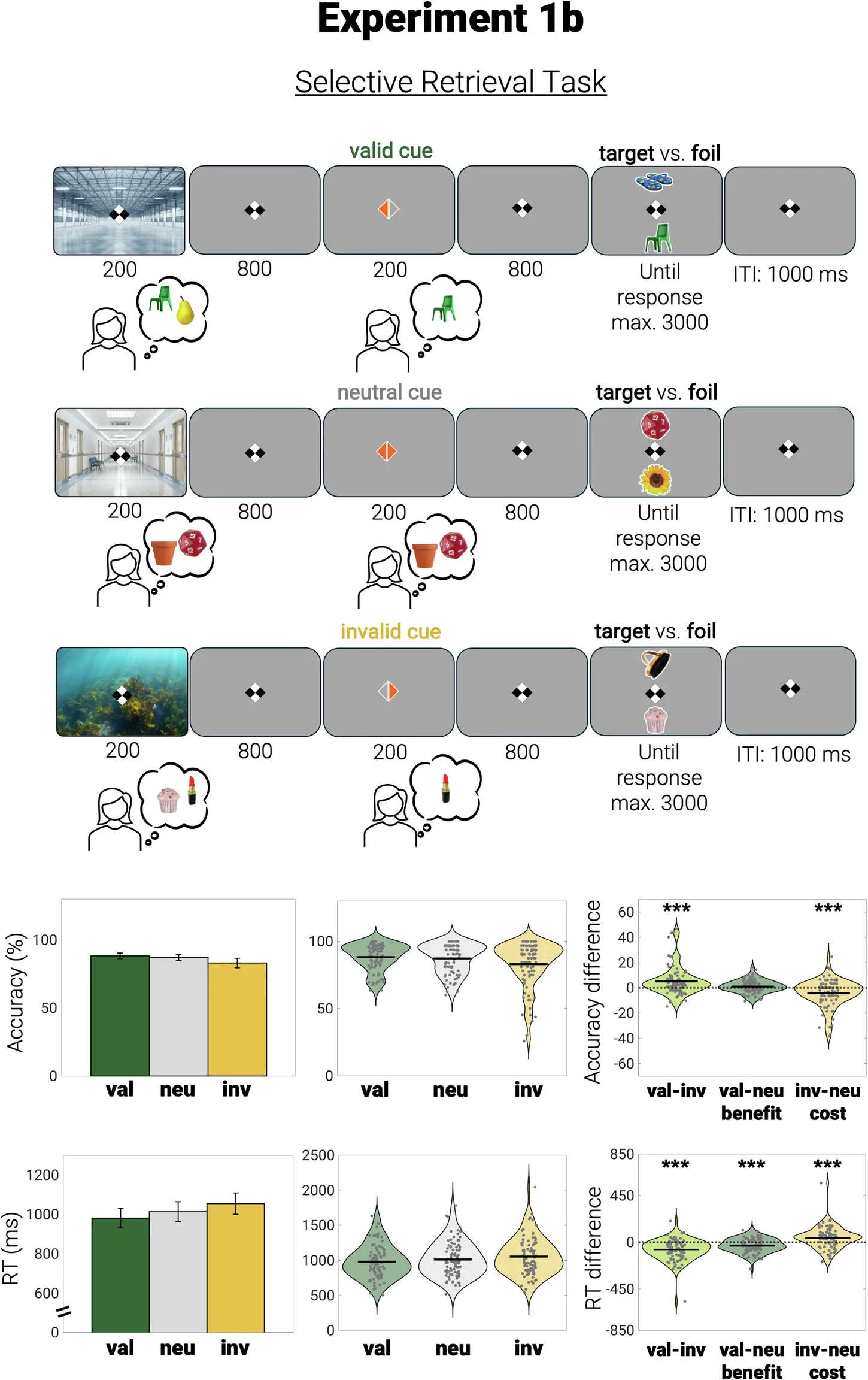
Selective Retrieval task design and results – Experiment 1b (*N* = 90). Top panel: experimental design. The Selective Retrieval task in Experiment 1b was identical to the one in Experiment 1a, except that the probe array contained a foil object associated with a different scene as the nontarget item. The bottom panel illustrates accuracy and response times results. We found attentional benefits reflected in accuracy. Additionally, participants were more accurate in the valid compared to the invalid trials. Response times results partially mirrored these effects. Results showed both attentional benefits and costs, as well as faster responses in the valid compared to the invalid trials. Both the bar plots and the violin plots (center) depict accuracy and response times contrasts between conditions, as indicated by the labels. Error bars in the bar plots represent the standard error of the mean. The right-side violin plots illustrate the three condition differences. The black solid line represents the condition means (middle panel) or the mean condition differences (right panel). Statistical significance is indicated with: *p < .05, **p < .01, ***p < .001.

Experiment 1b was designed to measure the putative benefits and costs of spatial retrocues in the context of an associative memory judgment. The details of the Selective Retrieval task in Experiment 1b were identical to those in Experiment 1a, except for the type of non-target object in the probe array (Fig. 3). Specifically, in Experiment 1b the probe array contained the target object, previously studied within the particular scene and another object studied within another scene (i.e., foil). Thus, during the probe, both the target and the non-target objects were equally familiar to the participant. Therefore, they could not rely on simple familiarity, and responses required the retrieval of the specific object-scene association.

##### Experiment 2

**Fig. 4:**
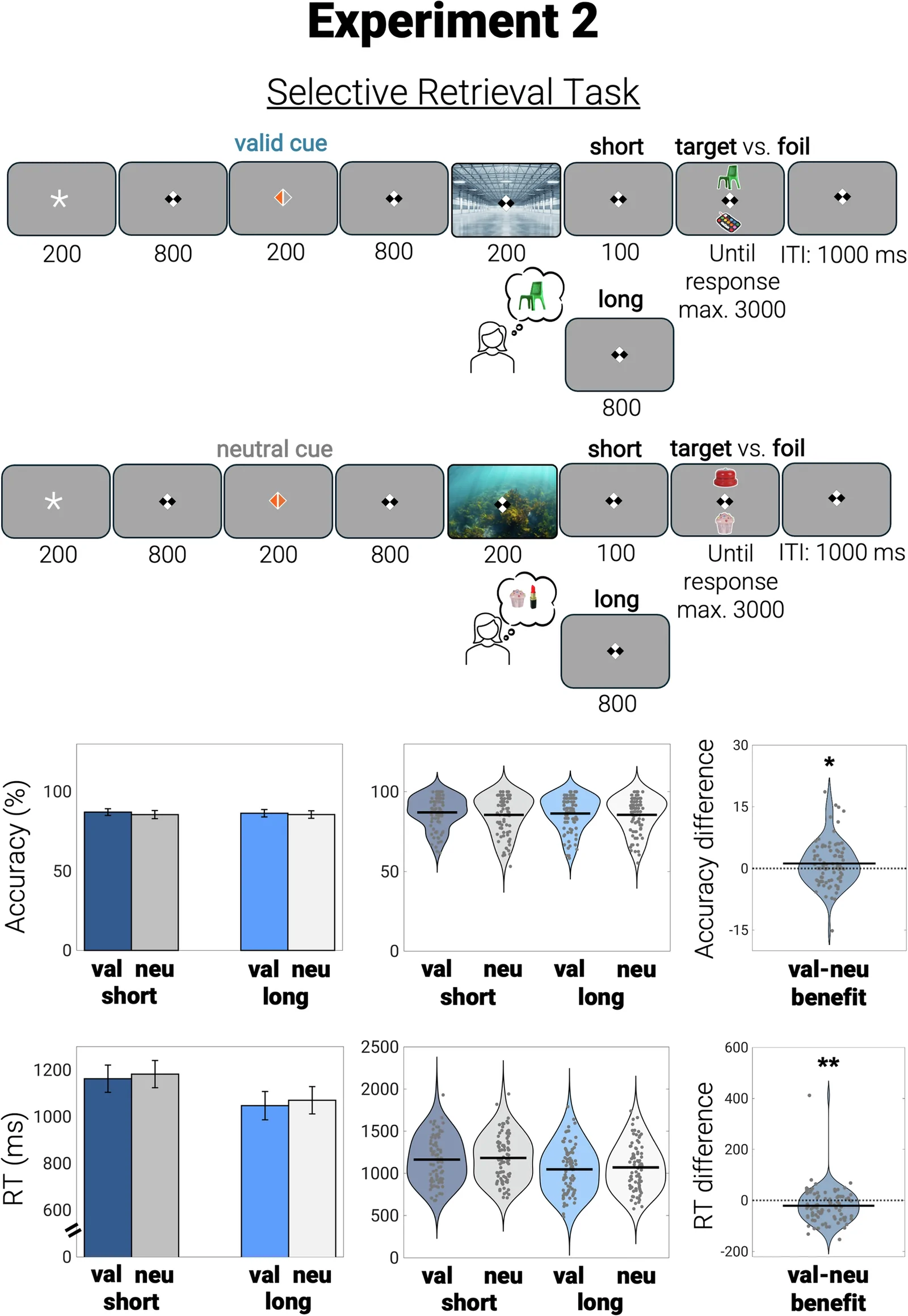
Selective Retrieval task design and results – Experiment 2 (*N* = 85). Top panel: experimental design. The Selective Retrieval task in Experiment 2 contained 100% informative and neutral cues. Since our aim was to test whether internal attention could impact the process of retrieving object associations before the scene and contextual memory could be uploaded into working memory, cues appeared before the scene presentation. Additionally, the retrieval interval was also varied: very short (100 ms) and long (800 ms). Each trial started with a star, indicating a new trial. After a short delay, a cue appeared on the screen, after which participants saw one of the previously learned scenes. The memory test was identical to Experiment 1b. Participants were shown a target object associated with the scene and a nontarget object, which was a foil linked to a different scene. The bottom panels illustrate accuracy and response times results. Accuracy results revealed only a main effect of precue: participants were more accurate in trials with a valid compared to a neutral cue. Response times analyses suggested both a main effect of precue and a main effect of retrieval interval, but no interaction effect. Specifically, participants were faster in valid vs. neutral trials, independent of retrieval delay. Both the bar plots and the violin plots (center) depict the accuracy and response times contrasts between conditions. Error bars in the bar plots represent the standard error of the mean. The right-side violin plots illustrate the valid-minus-neutral difference. The black solid line represents the condition means (middle panel) or the mean condition differences (right panel). Statistical significance is indicated with: *p < .05, **p < .01, ***p < .001.

The Selective Retrieval task of Experiment 2 aimed to test whether internal attention could confer retrieval benefits early in the retrieval process and under fast-paced conditions (Fig. 4). A valid or neutral cue preceded the presentation of the scene. Next, the scene was presented for 200 ms and the probe array followed after a shorter or longer interval, prompting the retrieval of a specific object-scene association (like in Experiment 1b). Experiment 2 did not include invalid cues. The decision to focus only the valid vs. neutral contrast was based on the significant attentional benefits in Experiment 1b. The motivation was to maintain a sufficient number of trials per condition after introducing the additional retrieval-interval manipulation.

Predictive cues appeared before the scene so that, in principle, selective attention could start to operate as soon as the retrieval of the scene-object association was initiated. Two retrieval-interval conditions were used. In one condition, participants had a long interval, 800 ms between the scene and the probe onset. In contrast, in the short condition (100 ms from scene offset to probe onset), the available time was strongly limited. We reasoned that the effect of valid cues would be smaller in the short-interval condition if the attentional modulation only starts after the objects are fully placed in working memory. In contrast, if modulation starts earlier in the retrieval process, both conditions will yield equivalent or similar attentional benefits.

Each trial began with a star (0.1 × 0.1 PsychoPy units) indicating trial onset. After an 800-ms delay, a precue appeared for 200 ms. The precue was either 100% spatially informative or neutral. Similar to Experiment 1, informative precues indicated the side on which the to-be-probed object had appeared (100% validity), whereas neutral precues provided no information. After an 800 ms delay, the scene appeared briefly (for 200 ms). Following the scene offset, a short (100 ms) or long (800 ms) interval preceded the appearance of the probe array. The probe array was similar to that in Experiment 1b. Objects appeared above and below fixation. The target object was previously associated with the target scene, whereas the non-target object was a foil previously associated with another scene. As in Experiments 1a and 1b, participants responded using the up or down arrow keys within a 3000-ms response window and were instructed to respond as quickly and as accurately as possible.

In each experimental session, participants completed two blocks of the Selective Retrieval task. In each block, the 48 object-scene associations were tested. Overall, across the two experimental sessions, participants completed 192 trials, with 48 trials within each condition (valid precue and short interval, valid precue and long interval, neutral precue and short interval, neutral precue and long interval). The assignment of each scene to a condition and the selection of the target and foil objects for each block were determined randomly.

### Analyses

Behavioral analyses were conducted in MATLAB® (R2023b), and statistical analyses were performed in JASP (v.0.19.3) and R (version 4.5.2)^69^. For both the Learning and Selective Retrieval tasks, the primary dependent variables were accuracy (% correct) and mean response times. Before conducting the main analyses, the data were cleaned. In line with the preregistration, trials with response times below 100 ms and those exceeding three standard deviations (Selective Retrieval task) were removed.

We examined learning across encoding blocks using a 2 × 4 repeated-measures ANOVA (rm-ANOVA) applied separately to accuracy and response times. The factors were block (blocks 1, 2, 4, 5) and test order (first and second probe). For the test blocks of the Learning task, localization performance was compared against chance level (25%), using one-sample t-ests (Supplementary materials, Tables S2 and S3).

For the Selective Retrieval tasks in Experiments 1a and 1b, rm-ANOVAs focused on the main effect of retrocue validity (valid, neutral, invalid retrocues) on accuracy and response times. In Experiment 2, we performed a rm-ANOVAs to test the main effect of precue (valid, neutral), the main effect of retrieval interval (short, long), and the precue x retrieval interval interaction. All reported analyses combined the two retrieval blocks.

#### Statistical analyses

We performed statistical analyses using both frequentist and Bayesian approaches. For the repeated-measures ANOVAs, the sphericity assumption was always tested. If sphericity was violated, the Greenhouse-Geisser correction was used. Partial eta squared (ηp²) was reported as the measure of effect size. The 95% confidence interval (CI) of the eta squared values was calculated using the *effectsize* package in R (version 1.0.2^70^. For t-tests, two-tailed *p*-values were calculated. Effect sizes and their 95% CI were computed using Cohen’s d (as implemented in JASP). When post-hoc comparisons were required, *p*-values were adjusted using the Holm method (as implemented in JASP). Corrected values are denoted as *p*_corr_.

Bayes factors were used to quantify the evidence for the alternative or null hypothesis. *BF*_*10*_ represents support for the alternative hypothesis, whereas *BF*_*01*_ (1/*BF*_*10*_) represents support for the null. Bayes factors below 3 indicate weak or inconclusive evidence, values between 3 and 20 indicate positive/substantial evidence, while the range 20–150 and above indicate strong and very strong evidence^71^. Both Bayesian rm-ANOVA and Bayesian t-tests were performed using a multivariate Cauchy prior as implemented in JASP.

## Results

All three experiments contained two separate tasks: a Learning task followed by a Selective Retrieval task.

### Effective learning of scene-object associations

Learning was highly effective across all experiments (Supplementary materials). The Learning task was the same in all experiments, with the exception that a larger set of scene-object associations was included in Experiment 2 (96 unique scenes with two objects) compared to Experiments 1a and 1b (80 unique scenes with two objects). Participants encoded the identities and locations of unique objects associated with scenes by performing an active working-memory task. To keep participants engaged, they reported each object’s location and received feedback (see Methods). Scene-object associations were encoded over multiple learning blocks, ensuring high levels of learning.

Participants’ performance in the active encoding task was good throughout all blocks (Supplementary materials). Mean accuracy was already high in the first block ( ~ 97% for all three experiments) and remained stable throughout (Supplementary materials; Tables S1–S2). We consistently observed a main effect of test order on accuracy: participants were more accurate in reporting the location of the second object than the first (Experiments 1a, 1b, and 2). Response-time results showed main effects of block and condition across all experiments, indicating that participants became faster across learning blocks and were quicker when reporting the second object’s location (Supplementary materials; Tables S1–S2). Overall, these findings indicate that participants were actively engaged in memorizing and reporting the scene-object associations.

Midway through the Learning task, a test block assessed the learning quality by asking participants to retrieve the location of each object from long-term memory. Accuracy in the test block of the Learning task significantly exceeded chance level (25%) in all three experiments (Supplementary materials; Table S3).

### Benefits of internal selective attention in contextual long-term memories

#### Experiment 1a

Experiment 1a tested for possible benefits and costs of internal spatial attention in the context of an old/new recognition judgment (Fig. 2). Experiment 1a used novel, previously unseen objects as the nontarget object in the probe array. Comparing performance following informative versus neutral retrocues in the Selective Retrieval task revealed both attentional benefits and costs, mainly reflected in response times. Data quality was good, with very few trials lost because of response timeouts (*M* = 1.05 trials; *SD* = 2.54).

Accuracy in the 2AFC test was close to ceiling ( > 95% overall) and showed no main effect of condition, *F*(2, 118) = 1.96, *p* = 0.14, η_*p*_^2^ = .03, 95% CI for the η_*p*_^2^ [0, 0.11], *BF*_10_ = 0.30 (inconclusive evidence). Response times were a more sensitive variable. They showed a robust main effect, supported by very strong evidence, *F*(2, 118) = 31.85, *p* < .001, η_*p*_^2^ = 0.35, 95% CI for the η_*p*_^2^ [0.21, 0.46], *BF*_10_ = 8.52×10^8^. Follow-up tests confirmed that responses were faster in valid compared to invalid trials, *t*(59)  =  −7.53, *p*_*corr*_ < .001, *d*  =  −0.30, 95% CI for Cohen’s d [−0.43, −0.18], 95% *CI* [−71.84, −36.43], *BF*_10_ = 3.11 × 10^7^. Comparisons with the neutral retrocue condition revealed both attentional benefits and costs. Specifically, responses were faster in valid compared to neutral retrocue trials, *t*(59)  =  −2.46, *p*_*corr*_ = .01, *d*  =  −0.08, 95% CI for Cohen’s d [−0.17, 0.002], 95% *CI* [−30.70, -0.01], *BF*_10_ = 2.27 (inconclusive evidence) and slower in invalid vs. neutral retrocue trials, *t*(59)  =  −5.17, *p*_*corr*_ < .001, *d*  =  -0.22, 95% CI for Cohen’s d [−0.33, −0.10], 95% *CI* [−57.25, −20.30], *BF*_10_ = 5828.73 (Table 2, Fig. 2).

**Table 2:** Descriptive statistics – Experiment 1a, *N* = 60

Condition	Mean	Standard error of the mean
Accuracy (%)		
valid	97.55	0.48
neutral	96.74	0.60
invalid	97.23	0.59
Response times (ms)		
valid	782.33	22.73
neutral	797.69	22.72
invalid	836.47	22.72

#### Experiment 1b

The Selective Retrieval task of Experiment 1b required participants to choose between an object previously associated with the particular scene and another previously studied foil linked to another scene. In all other respects, the task was identical to that in Experiment 1a. Both accuracy and response time measures revealed an effect of internal attention on retrieval performance. Data quality was good, with very few trials lost because of response timeouts (*M* = 1.24 trials; *SD* = 3.04).

Accuracy in the 2AFC test showed good memory performance that was not at ceiling. Numerically, accuracy was better in trials with valid ( ~ 88%) compared to invalid retrocues ( ~ 83%), with intermediate performance on neutral retrocue trials ( ~ 87%). The accuracy analyses revealed a main effect of condition, supported by very strong evidence, *F*_corr_(1.32, 118.06) = 15.05, *p*_corr_ < .001, η_p_^2^ = 0.14, 95% CI for the η_*p*_^2^ [0.04, 0.26], *BF*_10_ = 14870.77 (due to sphericity violation, χ^2^(2) = 62.36, *p*  <  .001, ε  =  0.66, Greenhouse-Geisser–corrected results are reported). Follow-up t-tests yielded very strong evidence that accuracy was higher in the valid relative to the invalid condition, *t*(89)  = 4.34, *p*_*corr*_ < .001, *d*  =  0.40, 95% CI for Cohen’s d [0.16, 0.64], 95% *CI* [2.30, 8.20], *BF*_10_ = 481.21. The accuracy benefit (valid vs. neutral) results were inconclusive, *t*(89)  =   1.90, *p*_*corr*_ = .06, *d*  =  0.08, 95% CI for Cohen’s d [-0.02, 0.18], 95% *CI* [−0.29, 2.37], *BF*_10_ = 0.65; whereas a robust attentional cost, supported by strong evidence, emerged for the invalid vs. neutral trials, *t*(89) =  3.66, p_corr_ < .001, *d*  =  0.32, 95% CI for Cohen’s d [0.10, 0.54], 95% CI [1.40, 7.01], *BF*_10_ = 50.81 (Table 3, Fig. 3).

**Table 3:** Descriptive statistics – Experiment 1b, *N* = 90

Condition	Mean	Standard error of the mean
Accuracy (%)		
valid	88.32	1.05
neutral	87.28	1.14
invalid	83.07	1.78
Response times (ms)		
valid	980.73	25.05
neutral	1013.40	25.83
invalid	1054.58	27.63

Analyses of response times similarly revealed a main effect of condition, with very strong evidence for the effect, *F*_corr_(1.62, 144.88) = 28.49, *p*_corr_ < .001, η_p_^2^ = 0.24, 95% CI for the η_*p*_^2^ [0.13, 0.35], *BF*_10_ = 4.26 × 10^8^ (due to sphericity violation, χ^2^(2) = 22.83, *p*  <  0.001, ε  =  0.81, Greenhouse-Geisser–corrected results are reported). Follow-up *t*-tests further confirmed that participants were faster in trials with valid vs. invalid retrocues, *t*(89) = −6.92, *p*_*corr*_ < .001, *d*  =  −0.29, 95% CI for Cohen’s d [−0.41, −0.17], 95% *CI* [−99.89, −47.81], *BF*_10_ = 1.51 × 10^7^. Response times were faster in the valid compared to the neutral retrocue trials, revealing strong attentional benefits, *t*(89) = −4.59, *p*_*corr*_ < .001, *d*  =  −0.13, 95% CI for Cohen’s d [−0.20, −0.05], 95% *CI* [−50.02, −15.32], *BF*_10_ = 1174.59. Additionally, participants were slower in invalid vs. neutral trials, indicating a robust attentional cost, *t*(89) = −3.69, *p*_*corr*_ < .001, *d*  =  −0.16, 95% CI for Cohen’s d [−0.27, −0.05], 95% *CI* [−68.35, −14.01], *BF*_10_ = 56.61 (Table 3, Fig. 3).

#### Experiment 1a vs. 1b

Experiment 1b was designed to have higher memory demands than Experiment 1a. Responses in Experiment 1b required the retrieval of specific scene-object associations and could not simply be based on familiarity. Therefore, it was expected that behavioral performance would decrease in Experiment 1b (see Table 4). A between-experiment rm-ANOVA with the factors cue condition (valid vs. invalid vs. neutral) and experiment (1a and 1b) was conducted to contrast the attentional effects across experiments. Accuracy results revealed a main effect of cue condition, *F*(2, 296) = 9.74, *p* < .001, η_p_^2^ = 0.06, 95% CI for the η_*p*_^2^ [0.02, 0.12], BF_10_ = 5424.22, a main effect of experiment, *F*(1, 148) = 48.40, *p* < .001, η_p_^2^ = 0.24, 95% CI for the η_*p*_^2^ [0.14, 0.36], BF_10_ = 6.45 × 10^7^, and a cue condition x experiment interaction, *F*(2, 296) = 9.34, *p* < .001, η_p_^2^ = 0.05, 95% CI for the η_*p*_^2^ [0.02, 0.12], BF_10_ = 7.24 × 10^13^. Analyses of response times yielded a main effect of cue condition, *F*(2, 296) = 47.23, *p* < .001, η_p_^2^ = 0.24, 95% CI for the η_*p*_^2^ [0.16, 0.32], BF_10_ = 9.64×10^16^, a main effect of experiment, *F*(1, 148) = 33.74, *p* < .001, η_p_^2^ = 0.18, 95% CI for the η_*p*_^2^ [0.09, 0.29], BF_10_ = 149191.01, but no cue condition x experiment interaction, *F*(2, 296) = 1.30, *p* = .27, η_p_^2^ = 0.009, 95% CI for the η_*p*_^2^ [0, 0.04], BF_10_ = 0.68. These results suggest that while the accuracy in Experiment 1a showed no modulation by attention, it was significantly impacted in Experiment 1b. Attentional effects on response times were comparable across experiments.

**Table 4:** Descriptive statistics for accuracy (%) - cue condition x experiment interaction

Condition	Experiment	*N*	Mean	Standard error of the mean
valid	1a	60	97.55	0.48
	1b	90	88.32	1.05
neutral	1a	60	96.74	0.60
	1b	90	87.28	1.14
invalid	1a	60	97.23	0.59
	1b	90	83.07	1.78

Descriptive statistics for response times (ms) - cue condition x experiment interaction				
valid	1a	60	782.33	22.73
	1b	90	980.73	25.05
neutral	1a	60	797.69	22.72
	1b	90	1013.40	25.83
invalid	1a	60	836.47	22.72
	1b	90	1054.58	27.63

#### Experiment 2

Experiment 2 explored further the possible benefits of attentional orienting during long-term memory retrieval. Informative or neutral precues appeared before the scene presentation to test whether internal attention could impact retrieval before the association could be uploaded into working memory. By using a very short (100 ms) and a longer (800 ms) retrieval interval, we sought to test whether internal attention acted directly upon the initial long-term retrieval or required more time, possibly involving working-memory mediation. Valid precues led to benefits in both accuracy and response times, and attentional benefits did not interact with the retrieval interval.

Similar to the 2AFC results in Experiment 1b, memory performance was high but not at ceiling. Furthermore, data quality was good, with very few trials lost because of response timeouts (*M* = 1.30 trials; *SD* = 2.54). The 2×2 rm-ANOVA conducted for accuracy revealed a main effect of precue, *F*(1, 84) = 4.06, *p* = .04, η_p_^2^ = 0.04, 95% CI for the η_*p*_^2^ [0, 0.16], *BF*_10_ = 0.90 (inconclusive evidence). The follow-up test further confirmed that participants were more accurate in the valid compared to the neutral condition, *t*(84)  =   2.01, *p*_corr_ = .04, *d*  =  0.10, 95% CI for Cohen’s d [1.77 × 10^-4^, 0.21], 95% *CI* [0.01, 2.39], *BF*_10_ = 1.18 (inconclusive evidence). We found no effect of retrieval interval, and the lack of this effect was supported by substantial evidence, *F*(1, 84) = 0.39, *p* = .53, η_p_^2^ = 0.005, 95% CI for the η_*p*_^2^ [0, 0.07], *BF*_01_ = 6.03. Finally, precue validity and retrieval interval did not interact, and the absence of interaction was supported by strong evidence, *F*(1, 84) = 0.52, *p* = .46, η_p_^2^ = 0.006, 95% CI for the η_*p*_^2^ [0, 0.08], *BF*_01_ = 30.99 (Table 5, Fig. 4).

**Table 5:** Descriptive statistics – Experiment 2, *N* = 85

Condition	Mean	Standard error of the mean
Accuracy (%)		
valid short	87.06	1.08
neutral short	85.48	1.30
valid long	86.35	1.19
neutral long	85.51	1.22
Response times (ms)		
valid short	1162.60	29.80
neutral short	1182.27	29.68
valid long	1047.27	30.95
neutral long	1070.37	29.79

Analyses of response times provided convergent evidence. The 2×2 rm-ANOVA revealed a main effect of precue, F(1, 84) = 7.94, *p* = .006, η_p_^2^ = 0.08, 95% CI for the η_*p*_^2^ [0.01, 0.22], BF_10_ = 6.36, and of retrieval interval, *F*(1, 84) = 349.37, *p* < .001, η_p_^2^ = 0.80, 95% CI for the η_*p*_^2^ [0.73, 0.85], *BF*_10_ = 3.16×10^28^. However, we found no evidence for an interaction effect, *F*(1, 84) = 0.13, *p* = 0.71, η_p_^2^ = 0.002, 95% CI for the η_*p*_^2^ [0, 0.06], *BF*_10_ = 0.59 (inconclusive evidence). Follow-up *t*-tests showed that participants responded more quickly in the valid than in the neutral precue condition, and this effect was supported by strong evidence, *t*(84)  =  −2.81, *p*_corr_ = .006, *d*  =  −0.07, 95% CI for Cohen’s d [−0.13, −0.02], 95% *CI* [−36.48, −6.29], *BF*_10_ = 21.28. Responses were also faster after long retrieval intervals compared to short retrieval intervals, and the effect was supported by very strong evidence, *t*(84)  =  18.69, *p*_corr_ < 0.001, *d*  =  0.41, 95% CI for Cohen’s d [0.33, 0.48], 95% *CI* [101.52, 125.70], *BF*_10_ = 3.99 × 10^45^(Table 5, Fig. 4).

## Discussion

In this proof-of-concept study, we introduced an effective experimental framework for investigating how selective attention operates on long-term memory contents. Building on the successful record of retrocueing studies developed to investigate attention to working memory contents^14,21^, we show that this experimental toolkit can also be successfully applied to long-term memory. Across three experiments, informative spatial retrocues (Experiments 1a and 1b) and precues (Experiment 2) significantly improved the retrieval of objects in 2AFC responses based on recognition (Experiment 1a) and recollection (Experiment 1b, Experiment 2).

### Main findings

Selective attention operating on contents from long-term memory modulated retrieval performance with magnitudes broadly consistent with the working-memory literature. Across all experiments, informative spatial cues directing attention within scene-object associations impacted response times. Specifically, relative to non-informative neutral retrocues, spatially predictive retrocues conferred attentional benefits when they validly indicated the associated object location and led to attentional costs when they indicated the wrong location (Experiments 1a and 1b). Informative precues, appearing before scene onset, similarly improved response times relative to non-informative precues (Experiment 2). The consistency of the response-time modulations, despite variations in the type of probe array and cue timing, speaks to the robustness of the experimental approach.

Accuracy effects depended on the nature of the required retrieval response. Spatially informative retrocues (Experiment 1b) or precues (Experiment 2) modulated accuracy when the retrieval task was more demanding and tapped into the specific scene-object association. In contrast, performance was very high when retrieval required simpler old/new judgments, which could make use of familiarity in addition to recollective processes^72,73^.

### Interpretations

The current study helps promote the view that attention functions are an integral part of selective retrieval^9,18^. The findings complement previous work done in this area^36,37,42,47,52,54–57,74^. Our experimental framework will help advance research on selective retrieval by offering a well-controlled and flexible means to isolate and manipulate attention. In our tasks, we separate the Learning and Selective Retrieval phases, which brings important advantages for studying internal attention in long-term memory. The separation of the Learning and Retrieval tasks allows for tight control over the quality of the associations. Many memory qualities may interact strongly with the ability of internal attention to select effectively between alternative contents. Thus, we believe our studies offer a promising platform for dissecting how selective attention can guide retrieval among competing contents within memory associations.

Having demonstrated the practicality and efficacy of using informative cues to guide retrieval, Experiment 2 manipulated the retrieval interval to gain initial insights into the time course of the attentional modulation during selective retrieval. The retrieval interval between the scene offset and probe onset was either 800 ms or 100 ms. Cueing benefits in response time and accuracy were preserved at the shorter interval, and we observed no interval × cue interaction. Our findings raise the intriguing possibility that selection and prioritization of long-term memory contents may start very early in the retrieval process, although alternative explanations remain open.

We considered that selection and prioritization could occur at three stages during retrieval: (1) early, during reactivation of the associated contents, but before being fully loaded into working memory; (2) after the contents were reactivated and placed into working memory; (3) or only at the very end of the trial when deciding between the options in the probe array. If attention functions can act early, before full reinstatement in working memory, benefits would be expected regardless of the retrieval interval (scenario 1). Our findings are compatible with such a scenario. In contrast, if long-term memory contents require full reinstatement into working memory (scenario 2), attentional benefits would be compromised with very short retrieval intervals. This was not observed, although reinstatement in working memory and prioritization could have continued to unfold after the memory probe until the final response was delivered. Finally, the findings are also compatible with attentional prioritization acting only later, to guide the decision-making process (scenario 3). Although the results do not provide definitive explanations, they leave open all possibilities, including one in which long-term memory retrieval is highly flexible and modulated by goals and expectations from the very first steps. We favor this highly flexible view of long-term memory retrieval, but future studies with time-resolved behavioral or neural measures will be required to arbitrate among the competing alternatives. It is also possible that attention-related modulations occur at more than one processing stage.

### Future directions

Our current experiments are just an initial step in a rich parameter space to be explored. Our experiments manipulated internal attention with cues pointing to the left or right. Spatial cues can be more specific than just hemifields. Other types of information can also be used to guide attention, such as object- or feature-related information or even higher-level attributes (e.g., relational, affective, or social aspects). Manipulating the type of informative cue (e.g., a centrally presented symbol, salient transient stimulus) may reveal distinct modulatory attention functions within retrieval. Varying the onset time of cues relative to the scene context and probe array will further help chart the dynamics of attention functions that can influence long-term memory. For example, multiple automatic and controlled modulatory processes may run in parallel and have different behavioral and neural consequences^75^. Furthermore, it will be exciting to extend the experimental approach to dynamic scene contexts^76^, in which it will also be possible to orient attention to the temporal attributes of events^77^.

Furthermore, it is possible to test how the attributes of the long-term memories interact with attention functions. For example, the degree of similarity between items associated with the same scene impacts their competition during encoding and retrieval^37,38,42^. By manipulating the degree and the type of item similarity (e.g., spatial proximity or sensory similarity), it will be interesting to test whether the strength of attention effects depends on the degree of competition, as it does in sensory^78,79^ and working-memory^80,81^ domains. In addition to the qualities of the associations, their strength and age may also matter. By using separate Learning tasks, the experimenter gains control over these parameters.

### Limitations

Despite its promise, our study has its limitations. The scope of the investigation was limited in two important respects. Only a narrow set of manipulations was performed, and measurements were confined to behavioral variables. The intention was to provide a solid foundation for future research seeking to investigate selective attention within long-term memory retrieval. The initial set of tasks was designed to test the consequences of attentional orienting to objects associated with scenes in long-term memory. Therefore, we did not test the granularity of the spatial information, nor whether attention can improve the quality of the retrieved associations. Future studies could manipulate the spatial proximity of the items learned within given scenes to test how the degree of spatial competition impacts selective-attention benefits for retrieval. Continuous report measures could also be used to probe the precision of spatial retrieval. Our 2AFC measure provides little insight into whether selective attention can improve the non-spatial qualities of the retrieved objects. However, responses using a continuous-response modality could provide evidence in this regard by using a color wheel to report the color of the retrieved object.

The behavioral nature of these proof-of-concept experiments precluded strong inferences about the mechanisms underlying the costs and benefits of selective attention. Although highly sensitive, the used 2AFC response does not provide detailed information regarding the types of decision variables that may be impacted (e.g., response criterion, certainty, and confidence, in addition to sensitivity). Furthermore, our dependent variables, accuracy and response times, sample the cognitive system only at one, late time point. Therefore, it is not possible to address unambiguously the question of whether attention operates at early or late stages during selective retrieval. By complementing these behavioral studies with neural measures with high temporal resolution (EEG, MEG), it would be possible to test whether selective attention impacts the retrieval process proactively, before the response probe, and, if so, how early in the retrieval process the modulation begins.

## Conclusion

Our experimental framework complements and extends the various strands of research investigating the role of attention in selective retrieval. The present findings vindicate theoretical accounts proposing a core, intrinsic role for selective attention in long-term memory retrieval^5,24–34,82^. The tasks complement research approaches that highlight competition among memories with similar or overlapping items or contexts^37,83–85^. They also complement studies of retrieval mode^86^ and retrieval orientation^50^ by enabling the investigation of the psychological functions and neural processes underlying memory retrieval and internal attention.

Our experimental approach thus paves the way for addressing many questions about long-term memory retrieval, some longstanding and others new. Fruitful topics include the types and time courses of selective attention functions; their dependence on intermediary working memory mediation; and their interaction with memory quality, strength, and competition. We suspect these tools will yield interesting insights into this rich and foundational intersection between memory and attention.

## Supplementary information

Transparent Peer Review file

Supplementary materials

## Data Availability

All data are available: https://doi.org/10.17605/OSF.IO/K9XU7.

## Code availability

All analyses scripts are available: https://doi.org/10.17605/OSF.IO/K9XU7.
