# Supplementary material for: Orienting selective attention within long-term contextual memories
Source: Commun Psychol. 2026 Jul 16;4:145. doi: 10.1038/s44271-026-00505-9 (PMC13690599; doi:10.1038/s44271-026-00505-9)
Supplement: Supplementary file 1 [file 44271_2026_505_MOESM1_ESM.pdf]

nature portfolio

Peer Review File

Orienting selective attention within long-term contextual memories

Corresponding Author: Professor Anna Nobre

This file contains all editorial decision letters in order by version, followed by all author rebuttals in order by version.

Version 0:

Decision Letter:

** Please ensure you delete the link to your author homepage in this e-mail if you wish to forward it to your coauthors **

Dear Professor Nobre,

Thank you for your patience during the peer-review process. Your manuscript titled "Selective Attention Within Contextual Long-Term Memory" has now been seen by 3 reviewers, and I include their comments at the end of this message. They find your work of interest but raised some important points. We are interested in the possibility of publishing your study in Communications Psychology, but would like to consider your responses to these concerns and assess a revised manuscript before we make a final decision on publication.

We therefore invite you to revise and resubmit your manuscript, along with a point-by-point response to the reviewers. Please highlight all changes in the manuscript text file.

Editorially, we consider it essential that you address the critiques regarding the theoretical interpretation of your findings. Specifically, you must rigorously distinguish your claim of "selective attention within LTM" from alternative explanations such as serial perceptual comparison and expectancy-driven retrieval preparation. Crucially, you must address the concern that the reaction time patterns in Experiment 2 appear to contradict the "direct access" hypothesis and instead support working memory mediation. Methodologically, please address the potential for response bias in your 2AFC accuracy metric (considering a signal detection approach if feasible) and clarify the potential testing effect confound in your block design. The introduction and discussion require revision to present a more coherent narrative that better aligns with what the behavioral data can support. And there are a number of requests for important methodological clarifications.

As you revise the manuscript in response to these issues, please also implement all requests in the attached Mandatory Revision Requests document. All requirements listed in this document need to be fully met, or the work will be returned to you for further revisions without peer review. This workflow is in place to increase the likelihood that the paper will be accepted for publication. It reduces the number of rounds of revision (and review) and ensures that the reviewers vet a version of the article that is compliant with journal policies. If you have any questions regarding the required revisions, please contact the journal prior to resubmission to avoid a negative outcome.

We are committed to providing a fair and constructive peer-review process. Please don't hesitate to contact us if you wish to discuss the revision in more detail.

Please submit the following items:

- Revised manuscript
- Point-by-point response to the referees' comments
- Mandatory Revision Requests Table (attached).
- Cover letter (as a separate document)

via this link: Link Redacted .

** This url links to your confidential home page and associated information about manuscripts you may have submitted or are reviewing for us. If you wish to forward this email to co-authors, please delete the link to your homepage first **

We hope to receive your revised paper within 8 weeks; please let us know if you aren't able to submit it within this time so that we can discuss how best to proceed. If we don't hear from you, and the revision process takes significantly longer, we may close your file. In this event, we will still be happy to reconsider your paper at a later date, provided it still presents a significant contribution to the literature at that stage.

We look forward to seeing the revised manuscript and thank you for the opportunity to review your work.

Best regards,

Troby Lui, on behalf of

Jesse Rissman

Troby Lui, PhD
Associate Editor
Communications Psychology

Jesse Rissman, PhD
Editorial Board Member
Communications Psychology
orcid.org/0000-0001-8889-5539

REVIEWER EXPERTISE:
Reviewer #1: long-term memory, working memory, attention
Reviewer #2: visual working memory, attention
Reviewer #3: visual working memory, cognitive control

REVIEWER REPORTS:

Reviewer #1 (Remarks to the Author):

This is an interesting study examining the role of selective attention on long-term memory (LTM) for objects and their locations within a scene. Three cleverly-designed experiments leveraged the retro-cue or pre-cue procedures common in studying selective attention in working memory to test the effects of such cuing on LTM recognition of objects. The results consistently show some effect of the cuing manipulation, either on accuracy (recognizing whether the object was in the scene, but only in the case of associative recognition) or RT. These are interesting findings.

I have a few criticisms of the paper in its current form:

(1) Given the 2 alternative forced choice (AFC) nature of the tests, it seems reasonable that accuracy suffices where one would typically like to see a model that isolates memory sensitivity from bias. At the same time, could bias still play a role in these tests? For example, there could be a bias toward the upper or lower response. One might assume this would "wash out," given that the correct response appears in the upper or lower location on half of the trials. But if there were an overall bias toward one of the responses, this could materially influence the obtained accuracy independent of actual memory sensitivity. At minimum, the authors must defend their choice of relying on accuracy as opposed to a potentially more pure metric of memory sensitivity.

(2) I was surprised to see no mention of previous studies of divided attention at retrieval. Such studies are ample and typically show effects either on accuracy or RT, in line with the idea that LTM retrieval is attentional demanding.

(3) There was a test phase in block 3 for object-scene pairings from the first two blocks. Were the items that were tested in this block also tested in the retrieval test occurring after the 5th block? If so, then those items had more opportunity for learning (e.g., testing effect) than did the items from the 4th and 5th blocks, and this should be accounted for in the analysis.

(4) I didn't understand the logic of Experiment 2, as to why the time manipulation (100ms vs 800ms) inherently tests whether selective attention in LTM "bypasses" working memory. The logic needs to be spelled out here, because as it stands, I'm not convinced the effect observed here rules out that internal attention in LTM requires mediation by working memory. In my view, LTM retrieval inherently requires working memory — the contents of the LTM must be brought to mind, which requires re-accessing those contents through working memory. What is the alternative?

(5) I couldn't help but think that a more powerful test for the selective attention to contents of a LTM representation would be to test using an AFC design the ability to discriminate the two items that had been in the scene, but in different locations. This would obviously only work with a valid retro-cue design, but essentially, the question becomes whether cuing to a specific location in the scene engages attention to the object that appeared there versus the other object that appeared (in a different location) in the same context. As it stands, the three experiments reported here kind of skirt around this question. In each of the experiments, there is no requirement to actually remember *where* in the scene the object appeared. There is merely the requirement to remember whether that object appeared in the scene *at all*.

Signed,
Nathaniel Greene

Reviewer #2 (Remarks to the Author):

The present study attempts to assess the role of selective attention in long-term memory (LTM) retrieval by putting forward a cueing paradigm that is adapted from studies of attentional priority in visual working memory (WM). Across three experiments, observers studied pairs of objects that were superimposed at unique locations on scenes. During later testing, observers were presented a studied scene and asked to complete a 2AFC recognition judgment to identify which object was originally presented in that scene. Prior to the 2AFC, observers received a spatial cue either after (E1A-B) or before (E2) the scene that indicated which object was likely to be tested with a high level of validity. Exp 1A-B showed that, relative to an uninformative cue, observers were faster at recognizing the target in the 2AFC when the cue was valid and slower when it was invalid, with mixed evidence for a cueing effect on accuracy. In Exp 2, the authors replicated the cueing benefit on RT and accuracy even when the pre-cued scene was followed by an extremely short ISI before test. The authors argue that their findings validate the utility of their paradigm for studying attentional selection in LTM and reveal cueing benefits that may circumvent mediation by WM.

The attempt to extend an existing experimental assessment of selective attention from WM to LTM is timely and deserves recognition. At the same time, I had some concerns about the present findings both in terms of their theoretical significance and their effectiveness at validating the task paradigm. Unless these concerns can be addressed through adequate revisions, I'm not sure the present findings substantially advance our understanding of selective attention in memory.

- My main concern is that many of the findings reported here do not seem to tell us a clear story about the role of selective attention in LTM retrieval. This jeopardizes both the theoretical novelty of the findings and the practical value of the task paradigm for isolating attentional selection in LTM. Here are some examples:
o The cueing effects on RT seem fully explained by the number of perceptual comparisons performed at test. When the cue is invalid, participants first perform two perceptual comparisons using the cued target before switching to the un-cued target and repeating this process (3-4 comparisons). When the cue is valid, there is no initial sequence of rejecting the probes because the cued target is always present in the test array (1-2 comparisons). The neutral condition then falls right in the middle because the observer searches for the present target first on half of the trials on average (2-3 comparisons). If this is a fair interpretation of the results, it would mean that cueing effects on RT are contained entirely within the perceptual comparison stage at test and would be expected to occur in a WM paradigm as well.
o The authors claim that the cueing benefits on RT in the short interval condition of E2 indicate that attention selected the cued target directly from LTM rather than waiting until both targets had been retrieved back into WM to initiate selection. The data seems to directly contradict such a conclusion. In Figure 4, there is a clear main effect of interval on RT where both the valid and neutral cues produced slower RTs in the short interval condition than the long interval condition. This is exactly what we would expect to happen if observers needed to wait until the targets were retrieved back into WM to apply attentional selection. Note also that the RTs in the long interval condition in E2 were similar to—if not slower than—the RTs in E1B, which suggests that presenting the cue before the scene did not facilitate any processing that occurred before or during scene onset. If this is also a fair interpretation of the results, then the findings of E2 merely replicate cueing effects in WM that have already been shown many times over.
o The cueing effect on accuracy was certainly intriguing, but the authors do not seem to provide a clear explanation for why this effect happened. For example, why did a cueing benefit on accuracy only occur in E2 and not E1B? Was it related to the timing of the spatial cue? The validity of the spatial cue? Was there a particular reason why the cue was rendered 100% valid only in E2?

- At a higher level, I felt that the clarity of the narrative could be improved in the Introduction and Discussion.
o The Introduction opens with the claim that knowledge about selective attention in LTM is “scant” before then proceeding to outline a large body of literature that never seems to build toward a coherent research question or thesis. When I finished the introduction, I wasn't sure what was going to be tested or shown beyond “benefits of orienting selective attention in long-term memory”.
o The Discussion describes a plethora of future directions that are related to attentional selection in LTM, but many of these directions do not seem uniquely realized or afforded by the task that the authors are proposing. For example, the authors spend a good deal of time highlighting how their learning task allows researchers to manipulate the age and similarity between LTM representations. Those types of manipulations are already quite common in LTM research, and it was not clear to me how the present paradigm might be superior to those previous approaches.

- I may have missed it, but can the authors provide some information about number of trials that were excluded for exceeding the 3000ms response window at test? Did these numbers differ at all between conditions or experiments?

Reviewer #3 (Remarks to the Author):

Summary: This three-experiment study aims to develop an experimental paradigm that supports the direct study of attention orienting in long-term memory (LTM). Participants in all three experiments first carried out a learning task in which they learned the pairings of scene contexts with two objects (each). Once having learned these pairings, participants' memories were probed in a task in using valid, invalid, and neutral retrocues in Experiments 1 & 2 and valid and neutral pre-cues in Experiment 3. Participants made 2AFC responses in each task selecting which of two items in a probe array is the target item. The results in general show a benefit of valid cueing and a cost of invalid cueing across the tasks. The authors conclude that these results support the use of this paradigm for further and deeper investigation of the mechanisms supporting selective attention from LTM.

Main comments:
In all, I find the manuscript to be methodologically sound. The use of scene–object associations and controlled cueing manipulations can provide a useful framework for examining how predictive information biases memory retrieval. However, the theoretical claims—particularly those concerning selective attentional orienting within LTM—appear to extend beyond what the current behavioral evidence can support. So, while I believe the experiments provide a solid demonstration that predictive cues bias retrieval from LTM, the current data more strongly support an account based on guided or selective retrieval than one invoking selective attentional orienting within LTM representations. Specifically, the manuscript frequently uses the term "selective attention" to describe the effects of informative cues on retrieval from LTM. Although this terminology is well-established in the context of working memory, it's unclear whether that is what is being measured here. The observed cueing benefits are consistent with selective retrieval or expectancy-based preparation and may not necessarily reflect attentional selection operating over competing LTM representations (see comments below). I think that clarifying whether the intended claim concerns attentional selection per se or selective retrieval more broadly would strengthen the theoretical framing and interpretation of the results.

Across the three experiments, the general finding is that valid cues tend to facilitate performance relative to neutral cues, and (when included) invalid cues incur costs. Although this pattern is reliable, it may also be a canonical consequence of predictive cueing as has been well-documented in both working memory and retrieval cueing literatures. Such effects can arise from strategic prioritization, expectancy-driven preparation, or decision-level bias (e.g., Souza & Oberauer, 2014; Posner, 1980; Günseli et al., 2015). Therefore, the results do not adjudicate among these possibilities and point specifically to selective attention operating over LTM representations.

An additional factor contributing to ambiguity in interpretation is the structure of the probe arrays. Participants are required to select the target object from a probe array also containing foil that is either novel or associated with a different scene. Even on invalidly cued trials, the probe array contains only a single object from the scene (the target) alongside an irrelevant foil, such that participants are not required to resolve competition between multiple concurrently relevant representations from the same long-term memory context. Consequently, performance can be optimized by prioritizing a single expected item rather than by selectively enhancing one representation while suppressing another. Although invalid trials demonstrate that participants can retrieve uncued information when the cue misleads them, this outcome reflects flexibility of retrieval following misleading cues rather than evidence for attentional selection operating simultaneously over multiple LTM representations.

The probabilistic cue validity manipulation in Experiments 1 and 2 (80% valid, 20% invalid) further encourages strategic retrieval preparation. Preparing for the cued object as the most likely response naturally produces both benefits on valid trials and costs on invalid trials, even in the absence of attentional modulation of memory representations. Therefore, I think the manuscript would benefit from more explicitly clarifying how such expectancy-based or decision-level mechanisms are distinguished from attentional orienting within LTM.

Experiment 3, while informative in demonstrating cue-driven facilitation, removes several features that could reveal selective attention. The absence of invalid cues eliminates reorienting demands and competitive selection, limiting the experiment's ability to speak to selective attention mechanisms. Although the cue-first design is interpreted as demonstrating attentional facilitation of LTM retrieval independent of working memory, it remains possible that the target object is loaded into working memory once the scene appears. The observation that response times are slightly faster following the longer delay is consistent with participants using this interval to pre-activate the relevant representation, suggesting that performance benefits may still be mediated via working memory maintenance rather than solely reflecting attentional selection over long-term memory.

Considering other performance patterns: Performance appears modestly worse in Experiments 2 and 3 relative to Experiment 1, with slower response times and slightly reduced accuracy, though no formal cross-experiment comparisons are reported. These patterns suggest increased task difficulty or inter-participant strategic variability without clear evidence of enhanced attentional selectivity. Moreover, accuracy does not vary across delay lengths in Experiment 3, consistent with effective maintenance of the relevant representation, while the slight speeding of response times at longer delays may reflect more efficient pre-activation rather than decay-resistant attention operating directly within LTM.

Minor comments:
i. The title in the manuscript file differs from what is in the online submission

ii. Were there any performance differences between Day 1 and Day 2? If learning carried over, then perhaps Day 2 effects would be stronger and more LTM-based?
iii. For clarification purposes: were there only four object locations or were locations more jittered than that? And were object locations within the scenes always left and right (meaning one object was always on the left and one always on the right)?
iv. The way that I understood the response 'grading', if the participant clicked on the correct quadrant, then the response was recorded as 'correct', otherwise, 'incorrect'. But, I wonder if there was a more continuous measure for accuracy, specifically, how far the click was from the actual location or were they just told to click on one of four quadrants?
v. Were there any performance differences for objects in the left part of the display versus the right? Or when the target probe was upper or lower? If participants used a particular and consistent scanning strategy, this could lead to behavioral differences according to these factors.
vi. An alternative explanation that came to mind is that participants may maintain/activate representations of both objects associated with a scene and use a comparison/search process at probe to identify the correct target. The observed cueing benefits and costs could then arise from biases in search order or retrieval readiness rather than from selective attentional orienting operating over LTM representations. Is there anything in the data that could address this or rule this out?

* TRANSPARENT PEER REVIEW: Communications Psychology uses a transparent peer review system. This means that we publish the editorial decision letters including Reviewers' comments to the authors and the author rebuttal letters online as a supplementary peer review file. However, on author request, confidential information and data can be removed from the published reviewer reports and rebuttal letters prior to publication. If your manuscript has been previously reviewed at another journal, those Reviewers' comments would not form part of the published peer review file.

** Visit Nature Research's author and referees' website at www.nature.com/authors for information about policies, services and author benefits**

Communications Psychology is committed to improving transparency in authorship. As part of our efforts in this direction, we are now requesting that all authors identified as 'corresponding author' create and link their Open Researcher and Contributor Identifier (ORCID) with their account on the Manuscript Tracking System prior to acceptance. ORCID helps the scientific community achieve unambiguous attribution of all scholarly contributions. You can create and link your ORCID from the home page of the Manuscript Tracking System by clicking on 'Modify my Springer Nature account' and following the instructions in the link below. Please also inform all co-authors that they can add their ORCIDs to their accounts and that they must do so prior to acceptance.
https://www.springernature.com/gp/researchers/orcid/orcid-for-nature-research

For more information please visit http://www.springernature.com/orcid

If you experience problems in linking your ORCID, please contact the Platform Support Helpdesk.

Version 1:

Decision Letter:

** Please ensure you delete the link to your author homepage in this e-mail if you wish to forward it to your coauthors **

Dear Professor Nobre,

Thank you for your patience during the peer-review process. Your manuscript titled "Orienting selective attention within long-term contextual memories" has now been seen by 3 reviewers, and I include their comments at the end of this message. They find your work of interest but raised some important points. We are interested in the possibility of publishing your study in Communications Psychology, but would like to consider your responses to these concerns and assess a revised manuscript before we make a final decision on publication.

We therefore invite you to revise and resubmit your manuscript, along with a point-by-point response to the reviewers. Please highlight all changes in the manuscript text file.

From an editorial perspective, the key priority for revision is to engage more directly with the remaining concerns about interpretation and theoretical scope. In particular, we encourage you to: (1) more clearly position the contribution of the present findings relative to prior work on attentional prioritization in working memory, (2) address alternative accounts based on post-retrieval comparison processes, and (3) clarify the assumptions underlying the interpretation of Experiment 2, especially with respect to claims about mediation by working memory. We do not view these issues as requiring new data, but rather a clearer and more balanced framing of the existing results.

We are committed to providing a fair and constructive peer-review process. Please don't hesitate to contact us if you wish to

discuss the revision in more detail.

Please submit the following items:

- Revised manuscript
- Point-by-point response to the referees' comments
- Mandatory Revision Requests Table (attached).
- Cover letter (as a separate document)

via this link: Link Redacted .

** This url links to your confidential home page and associated information about manuscripts you may have submitted or are reviewing for us. If you wish to forward this email to co-authors, please delete the link to your homepage first **

We hope to receive your revised paper within 8 weeks; please let us know if you aren't able to submit it within this time so that we can discuss how best to proceed. If we don't hear from you, and the revision process takes significantly longer, we may close your file. In this event, we will still be happy to reconsider your paper at a later date, provided it still presents a significant contribution to the literature at that stage.

We look forward to seeing the revised manuscript and thank you for the opportunity to review your work.

Best regards,

Troby Lui, on behalf of

Jesse Rissman

Troby Lui, PhD
Associate Editor
Communications Psychology

Jesse Rissman, PhD
Editorial Board Member
Communications Psychology
orcid.org/0000-0001-8889-5539

REVIEWER REPORTS:

Reviewer #1 (Remarks to the Author):

The authors have responded thoroughly to my concerns on their previous submission. I believe this version of the manuscript is much improved, and I have no further comments.

Reviewer #2 (Remarks to the Author):

The authors have made significant revisions to their manuscript which have greatly improved the clarity of their arguments and tightened the theoretical focus. However, I'm still not strongly convinced that this study is making a substantive contribution that moves beyond what we've already gained from studies of attentional priority in WM. The focus on LTM is well-motivated, but the results only appear to replicate to what has already been shown in WM studies now using retrieved LTMs instead of recent percepts. The authors do attempt to produce a theoretical advance that is unique to LTM in E2, but it seems that the authors and I both agree that the evidence is not compelling enough to truly challenge existing theory.

Below, I outline my concerns in detail to have them on the record as part of my review. However, I'm happy to defer my recommendation to the discretion of the editor and the consensus of the other peer reviewers. If these folks feel that the findings reported in this current version of the manuscript are substantive enough to warrant publication, then the paper should be moved forward.

1. I raised the possibility that the cueing effects observed could be explained by the number of perceptual comparisons performed at test. My original intent was not to challenge the claim that people prioritize one of the two items prior to the

report (though I can see how this was not clear from my original comments). On the contrary, prioritizing the cued item prior to the report is what ensures that it is used for comparison first. Instead, my point was that, if the effect of priority on accuracy and RT can be reduced to the number of comparisons performed at test—or any other process that occurs *after reinstatement into WM*—then the cueing effects reported here do not demonstrate an influence by attention that was *unique to LTM* and differs in a *meaningful way* from effects already shown in WM studies.

So, can these results be explained by differences in the number of comparisons? The authors claim that the number of comparisons made at test can account for cueing effects on RT but not on accuracy, but no rationale is provided for the latter claim. On the contrary, there is ample evidence showing that memory probes induce interference that is greater in de-prioritized items than prioritized items (see, e.g., Makovski et al., 2008 JEPLMC; Saito et al., 2025 JEP:G). Therefore, it seems perfectly plausible that performing 1-2 additional probe comparisons (1) slows reports and (2) makes them slightly less accurate.

2. I and another reviewer raised the concern that the findings in E2 do not challenge the assumption of mediation by WM in LTM retrieval. We based this concern on the main effect of interval in RT, which showed that reports were delayed in the short interval condition compared to the long interval condition across cues, which would be expected if observers had to wait until retrieval into WM was complete to prioritize memoranda. The authors claim that mediation by WM should have produced an interaction between interval and cue validity, where cueing benefits in RT and accuracy are weaker in the short interval condition than the long interval condition. Much like their claim described in comment #1, the rationale for this claim is never clearly articulated. Based on what was provided, the authors seem to be saying that retrieval is slow, so when the SOA is extremely short, people should not have enough time to recall both items into WM and prioritize one of them before the report probes onset. As a result, on some trials prioritization never actually happens, which should reduce the size of the cueing benefit when averaging across trials. To make sense of the main effect of interval on RT, the authors suggest that the delay could be explained by differences in motor readiness or response preparation.

Critically, the only way that this interaction can be used as grounds to test for mediation by WM is if all of the assumptions above are true, but no evidence is provided to substantiate any of them. As such, it remains equally tenable that items were retrieved into WM and prioritized even after the report probes onset, which produced a comparable cueing benefit in the short condition that was slightly delayed relative to the long condition. Of course, the authors are welcome to continue taking a side on this issue in their General Discussion, but they should not unfairly ground the issue on an interaction that may not actually be diagnostic of mediation by WM.

Reviewer #3 (Remarks to the Author):

The authors satisfactorily addressed my previous comments. The revised narrative, in particular, improves the flow of the manuscripts and the framing of the study. The other clarifications in response to all of the comments raised by the reviewers makes clear the aims of the study and properly situates the results in the literature.

* TRANSPARENT PEER REVIEW: Communications Psychology uses a transparent peer review system. This means that we publish the editorial decision letters including Reviewers' comments to the authors and the author rebuttal letters online as a supplementary peer review file. However, on author request, confidential information and data can be removed from the published reviewer reports and rebuttal letters prior to publication. If your manuscript has been previously reviewed at another journal, those Reviewers' comments would not form part of the published peer review file.

** Visit Nature Research's author and referees' website at www.nature.com/authors for information about policies, services and author benefits**

Communications Psychology is committed to improving transparency in authorship. As part of our efforts in this direction, we are now requesting that all authors identified as ‘corresponding author’ create and link their Open Researcher and Contributor Identifier (ORCID) with their account on the Manuscript Tracking System prior to acceptance. ORCID helps the scientific community achieve unambiguous attribution of all scholarly contributions. You can create and link your ORCID from the home page of the Manuscript Tracking System by clicking on ‘Modify my Springer Nature account’ and following the instructions in the link below. Please also inform all co-authors that they can add their ORCIDs to their accounts and that they must do so prior to acceptance.
https://www.springernature.com/gp/researchers/orcid/orcid-for-nature-research

For more information please visit http://www.springernature.com/orcid

If you experience problems in linking your ORCID, please contact the Platform Support Helpdesk.

Version 2:

Decision Letter:

** Please ensure you delete the link to your author homepage in this e-mail if you wish to forward it to your coauthors **

Dear Professor Nobre,

Your manuscript titled "Orienting selective attention within long-term contextual memories" has now been assessed editorially. I am delighted to say that we are happy, in principle, to publish a suitably revised version in Communications Psychology.

We therefore invite you to revise your paper one last time to address the remaining concerns of our reviewers and a list of editorial requests. At the same time we ask that you edit your manuscript to comply with our format requirements and to maximise the accessibility and therefore the impact of your work.

EDITORIAL REQUESTS:

Please review our specific editorial comments and requests regarding your manuscript in the attached "Editorial Requests Table". Please outline your response to each request in the right hand column. Please upload the completed table with your manuscript files as a Related Manuscript file.

If you have any questions or concerns about any of our requests, please do not hesitate to contact me.

SUBMISSION INFORMATION:

In order to accept your paper, we require the files listed here https://www.nature.com/documents/commsj-file-checklist.pdf .

OPEN ACCESS:

Communications Psychology is a fully open access journal. Articles are made freely accessible on publication. For further information about article processing charges, open access funding, and advice and support from Nature Research, please visit https://www.nature.com/commspsychol/open-access

At acceptance, you will be provided with instructions for completing the open access licence agreement on behalf of all authors. This grants us the necessary permissions to publish your paper. Additionally, you will be asked to declare that all required third party permissions have been obtained, and to provide billing information in order to pay the article-processing charge (APC).

* TRANSPARENT PEER REVIEW: Communications Psychology uses a transparent peer review system. On author request, confidential information and data can be removed from the published reviewer reports and rebuttal letters prior to publication. If you are concerned about the release of confidential data, please let us know specifically what information you would like to have removed. Please note that we cannot incorporate redactions for any other reasons.

* CODE AVAILABILITY: All Communications Psychology manuscripts must include a section titled "Code Availability" at the end of the methods section. We require that the custom analysis code supporting your conclusions is made available in a publicly accessible repository at this stage; please choose a repository that generates a digital object identifier (DOI) for the code; the link to the repository and the DOI must be included in the Code Availability statement. Publication as Supplementary Information will not suffice.

* DATA AVAILABILITY:
All Communications Psychology manuscripts must include a section titled "Data Availability" at the end of the Methods section. More information on this policy, is available in the Editorial Requests Table and at http://www.nature.com/authors/policies/data/data-availability-statements-data-citations.pdf.

Please use the following link to submit the above items:
Link Redacted
** This url links to your confidential home page and associated information about manuscripts you may have submitted or be reviewing for us. If you wish to forward this email to co-authors, please delete the link to your homepage first **

We hope to hear from you within two weeks; please let us know if you need more time.

Best regards,

Troby Lui

Troby Lui, PhD
Associate Editor
Communications Psychology

Jesse Rissman, PhD
Editorial Board Member
Communications Psychology
orcid.org/0000-0001-8889-5539

** Visit Nature Research's author and referees' website at www.nature.com/authors for information about policies, services and author benefits**

Open Access This Peer Review File is licensed under a Creative Commons Attribution 4.0 International License, which permits use, sharing, adaptation, distribution and reproduction in any medium or format, as long as you give appropriate credit to the original author(s) and the source, provide a link to the Creative Commons license, and indicate if changes were made.
In cases where reviewers are anonymous, credit should be given to 'Anonymous Referee' and the source.
The images or other third party material in this Peer Review File are included in the article's Creative Commons license, unless indicated otherwise in a credit line to the material. If material is not included in the article's Creative Commons license and your intended use is not permitted by statutory regulation or exceeds the permitted use, you will need to obtain permission directly from the copyright holder.
To view a copy of this license, visit https://creativecommons.org/licenses/by/4.0/

Replies to reviewers' comments:

We thank the reviewers for their deep engagement with the study and manuscript and their thoughtful and constructive comments. The points raised were extremely helpful in pinpointing areas that lacked clarity and interpretations that were not fully convincing. Thank you also for asking for additional contextualization, methodological details, and further analyses. We believe the revised manuscript benefited substantially from the reviewers' input. Below, we address some common issues raised by the reviewers, followed by the additional points raised by each reviewer in turn.

We include the reviewers' comments in black font and provide our replies in light blue. To facilitate the reviewing process, we transcribed the core changes in the text in *italicized blue text.*

1. Common issues raised by the reviewers:

In different ways, all reviewers requested greater clarifications regarding the motivations and competing hypotheses that inspired our Experiment 2. It is helpful for us to know that our logic was not sufficiently spelled out or clear to informed readers. This is a key point for our study, and its reasoning is worth expanding.

We understand that the standard view is that long-term memory retrieval involves working memory mediation. (Although, interestingly, we note that most long-term memory-focused research does not often address this issue directly.) In our view, there are a few different possibilities.

(1) Standard: During long-term memory retrieval, all the contents associated with an event are placed in working memory. Therefore, internal selective attention mechanisms select and prioritize items within working memory to improve long-term memory retrieval performance.

(2) Alternative: attention functions that select and prioritize content operate very early on within the retrieval process. This is supported by the idea that brain regions involved in associative/contextual long-term memories (e.g., in the medial temporal lobe) have direct feedback connections to sensory areas in addition to connections to working memory areas (in parietal and prefrontal cortex). According to this alternative, by the time the content is transferred into working memory, it already underwent attentional modulation.

(3) Another possibility: Spatial cues are only used at the probe onset to guide the decision. This implies that no attentional modulation occurs either early in the retrieval process or at the time when the contents are transferred to working memory. We think this is unlikely, but it is possible.

Our findings tentatively favor the second option, but we concede that behavioral findings alone are inconclusive. Having established the behavioral methodology, we and other researchers

will be able to follow up with neural measures that can track the time course of how contents are modulated during long-term memory retrieval.

To spell out the logic of our study more clearly, we have added the following text to the revised manuscript.

> *"In principle, the selection and prioritization of memory contents might operate at three different stages (1) Long-term memory retrieval may necessarily involve mediation via working memory. Selection and prioritization may occur only after the long-term memory contents have been transferred to working memory, acting in much the same way as internal attention in working memory for recently encoded contents. (2) Alternatively, attention functions may start modulating long-term memory contents very early on before the representations are transferred to working memory. This is enabled by the existence of functional anatomical connections between long-term memory storage regions (e.g., in the medial temporal) and visual regions required for retrieving the sensory qualities of the associated contents. In this scenario, selection and prioritization of contents may occur before reaching an active state in working memory involving prefrontal and parietal areas. (3) A final possibility is that attention functions do not impact the retrieval process itself, but instead operate later, at the response stage. In this case, attentional processes may help guide the decision of which retrieved content is relevant or correct. These three possibilities are not mutually exclusive and there could be hybrid or other variations.*
>
> *By using long (1000 ms) or short (300 ms) retrieval intervals, we changed how much time participants had for engaging attention functions during retrieval. Long intervals afforded time to retrieve contents into working memory, but this ability was likely compromised in the short-interval condition. We hypothesized that, if selective attention only acts after the long-term memory content is in working memory (scenario 1), its benefits should be stronger with a long retrieval interval than with a short one. Alternatively, if attention functions can operate from the very first steps in the retrieval process (scenario 2), their benefits would also be evident with short retrieval intervals. Therefore, cueing effects should be comparable irrespective of the retrieval interval, so no cueing x retrieval interval interaction is expected in this case. The pattern of results would be inconclusive for scenario 3." (page 6)*
>
> —
>
> *"Predictive cues appeared before the scene so that, in principle, selective attention could start to operate as soon as the retrieval of the scene-object association was initiated. Two retrieval-interval conditions were used. In one condition, participants had a long interval, 1000 ms between scene and probe onsets. In contrast, in the short condition (300 ms from scene onset to probe onsets), the available time was strongly limited. We reasoned that the effect of valid cues would be smaller in the short-interval condition if the attentional modulation only starts after the objects are fully placed in working memory. In contrast,*

if modulation starts earlier in the retrieval process, both conditions would yield equivalent or similar attentional benefits." (page 11)

—

Experiment 2 manipulated the retrieval interval to gain initial insights into the time course of attention modulation during selective retrieval. Our findings raise the intriguing possibility that selection and prioritization of long-term memory contents may start very early in the retrieval process. The brief exposure to the scene stimulus triggers the retrieval process, typically assumed to be mediated by working memory. We considered that selection and prioritization could occur at three stages: (i) early during reactivation of the associated contents and before they were fully established within working memory; (ii) after the contents were reactivated and placed in working memory; (iii) or only at the very end of the trial when deciding between the options in the probe array. Assuming that placing contents in working memory takes time, short retrieval intervals should diminish the benefits of attention functions that operate within working-memory representations. Our results showed significant and equivalent benefits of attention with short or long retrieval intervals. The findings are compatible with selection and prioritization happening from the earliest stages of retrieval or, instead, only at latest stage when making the 2AFC response. The former possibility points to a highly flexible retrieval process, modulated by goals and expectations from the first steps. The latter points to a more rigid retrieval process, with no flexible modulation until the individual is provided with possible retrieval outcomes. We favor the former possibility, for two main reasons. In everyday situations, successful selective retrieval operates without us being presented with outcome options and having to decide among all possible alternatives. Furthermore, previous studies using brain imaging have clearly highlighted goal-related modulation of memory representations during retrieval before the presentation of response arrays (e.g. Kuhl et al., 2012). However, we acknowledge that future studies incorporating neural measures into our designs will be better equipped to track the stages of modulation. It is possible, and indeed likely, that attention-related modulation may occur at more than one processing stage." (page 19)

2. Replies to Reviewer #1

This is an interesting study examining the role of selective attention on long-term memory (LTM) for objects and their locations within a scene. Three cleverly-designed experiments leveraged the retro-cue or pre-cue procedures common in studying selective attention in working memory to test the effects of such cuing on LTM recognition of objects. The results consistently show some effect of the cuing manipulation, either on accuracy (recognizing whether the object was in the scene, but only in the case of associative recognition) or RT. These are interesting findings.

We thank Dr. Greene for the positive evaluation of our work and for the constructive and thoughtful comments. These have greatly helped us spell out our premises and motivations more clearly, as well as provide stronger evidence for the findings.

I have a few criticisms of the paper in its current form:

1) Given the 2 alternative forced choice (AFC) nature of the tests, it seems reasonable that accuracy suffices where one would typically like to see a model that isolates memory sensitivity from bias. At the same time, could bias still play a role in these tests? For example, there could be a bias toward the upper or lower response. One might assume this would "wash out," given that the correct response appears in the upper or lower location on half of the trials. But if there were an overall bias toward one of the responses, this could materially influence the obtained accuracy independent of actual memory sensitivity. At minimum, the authors must defend their choice of relying on accuracy as opposed to a potentially more pure metric of memory sensitivity.

   As the reviewer implies, the 2AFC method is less sensitive to decision biases than other types of recall judgments, like deciding whether an object is old/new. We chose the 2AFC method first of all for its greater sensitivity to memory traces. Second, since it is a relative response between old x new items, it overcomes the difficult criterion/confidence-related issues. We understand the benefit of explaining the rationale for using a 2AFC response (which was also what we pre-registered) and have included this in the Methods section of the revised paper.

   > *"The 2AFC method was chosen for its greater sensitivity to the presence of memory traces, the reduced impact of changes in response criteria, and its higher statistical power in distinguishing target vs. non-target memory distributions." (page 10)*

   The reviewer is correct that, in principle, there could be a bias for choosing upper x lower visual field items. This would not impact the results, since old x new/foil items were fully balanced in the response arrays. Nevertheless, just to be sure, we checked for such position biases in our data. Results indicated no significant impact of target vs. non-target position in any of the experiments. In all cases, the Bayes factor supported the null hypothesis:

   - Experiment 1a: $t(59) = -0.23$, $p = 0.81$, $BF_{01} = 6.90$.
   - Experiment 1b: $t(89) = -1.15$, $p = 0.25$, $BF_{01} = 4.51$.
   - Experiment 2 : $t(84) = 0.92$, $p = 0.36$, $BF_{01} = 5.54$.

2) I was surprised to see no mention of previous studies of divided attention at retrieval. Such studies are ample and typically show effects either on accuracy or RT, in line with the idea that LTM retrieval is attentional demanding.

Thank you for pointing us to this related literature. Our objective was to investigate selective attention operating during long-term memory retrieval operations rather than to examine the extent to which retrieval is impacted by competing task demands more generally. Nevertheless, the reviewer is right that this complementary line of research enriches and provides important context to the current work.

We have included new sections on divided attention at retrieval in both the Introduction and the Discussion.

"For example, work investigating divided attention during long-term memory retrieval suggested that competing attentional demands from concurrent tasks or perceptual distractors impair retrieval performance (Ataseven et al., 2023; Craik, 2021; Craik et al., 1996, 2018; Fernandes & Moscovitch, 2000; Rohrer & Pashler, 2003). Thus, divided-attention manipulations show that retrieval operations involve attentional resources." (page 3)

"The present findings vindicate theoretical accounts proposing a core, intrinsic role for selective attention in long-term memory retrieval (Aly & Turk-Browne, 2017; Ataseven et al., 2023; Cabeza et al., 2008; Chun & Johnson, 2011; Ciaramelli et al., 2008; Craik, 2021; Craik et al., 1996, 2018; Fernandes & Moscovitch, 2000; Hutchinson et al., 2009; Hutchinson & Turk-Browne, 2012; Madore & Wagner, 2022; Rohrer & Pashler, 2003)." (page 20)

3) There was a test phase in block 3 for object-scene pairings from the first two blocks. Were the items that were tested in this block also tested in the retrieval test occurring after the 5th block? If so, then those items had more opportunity for learning (e.g., testing effect) than did the items from the 4th and 5th blocks, and this should be accounted for in the analysis.

Thank you for raising this point, which helps us clarify our methodology. All associations were part of the Test Block (Block 3) of the Learning Task. Similarly, all associations were trained in the four blocks of the Learning Task (Blocks 1, 2, 4, 5). This means that all associations were on similar footing at the time of the Selective Retrieval Task. We have made the procedure clearer in the revised manuscript.

"Each learning block included training on all scenes and both objects associated with the respective scene. This ensured equal exposure to all associations." (page 9)

"All scenes-object associations were tested in the Test block." (page 9)

We would also like to point out that the purpose of the Test Block (Block 3, Learning Task) was to strengthen the object-scene associations before the final test in the Selective

Retrieval Task. The nature of the test in Block 3 differed from the one in the Selective Retrieval Task. In Block 3, participants had to actively report the object's location. In contrast, the Selective Retrieval Task used a 2AFC procedure. Thus, the response format and retrieval demand differed substantially, so any testing effect from Block 3 would not selectively benefit one condition in the final task.

4) I didn't understand the logic of Experiment 2, as to why the time manipulation (100ms vs 800ms) inherently tests whether selective attention in LTM "bypasses" working memory. The logic needs to be spelled out here, because as it stands, I'm not convinced the effect observed here rules out that internal attention in LTM requires mediation by working memory. In my view, LTM retrieval inherently requires working memory — the contents of the LTM must be brought to mind, which requires re-accessing those contents through working memory. What is the alternative?

   Thank you for pointing out that our logic was not transparent. We address this point in our replies to the common points raised by the reviewers.

5) I couldn't help but think that a more powerful test for the selective attention to contents of a LTM representation would be to test using an AFC design the ability to discriminate the two items that had been in the scene, but in different locations. This would obviously only work with a valid retro-cue design, but essentially, the question becomes whether cuing to a specific location in the scene engages attention to the object that appeared there versus the other object that appeared (in a different location) in the same context. As it stands, the three experiments reported here kind of skirt around this question. In each of the experiments, there is no requirement to actually remember *where* in the scene the object appeared. There is merely the requirement to remember whether that object appeared in the scene *at all*.

   This would be an interesting follow-up. Our research question was whether it is possible to selectively prioritize certain items within a learned context and how this affects item retrieval. Indeed, we did not aim to test the quality of the spatial memory. However, we know that participants did encode and retain spatial information from the fact that spatially informative cues conferred behavioral benefits.

   The reviewer's experimental idea should be feasible. Internal attention studies using continuous report measures in working memory show that informative cues can improve the report of item features and locations. This could be adapted to long-term memory designs, but it would address a different (the next?) set of questions.

Signed,
Nathaniel Greene

3. Replies to Reviewer #2:

The present study attempts to assess the role of selective attention in long-term memory (LTM) retrieval by putting forward a cueing paradigm that is adapted from studies of attentional priority in visual working memory (WM). Across three experiments, observers studied pairs of objects that were superimposed at unique locations on scenes. During later testing, observers were presented a studied scene and asked to complete a 2AFC recognition judgment to identify which object was originally presented in that scene. Prior to the 2AFC, observers received a spatial cue either after (E1A-B) or before (E2) the scene that indicated which object was likely to be tested with a high level of validity. Exp 1A-B showed that, relative to an uninformative cue, observers were faster at recognizing the target in the 2AFC when the cue was valid and slower when it was invalid, with mixed evidence for a cueing effect on accuracy. In Exp 2, the authors replicated the cueing benefit on RT and accuracy even when the pre-cued scene was followed by an extremely short ISI before test. The authors argue that their findings validate the utility of their paradigm for studying attentional selection in LTM and reveal cueing benefits that may circumvent mediation by WM.

The attempt to extend an existing experimental assessment of selective attention from WM to LTM is timely and deserves recognition. At the same time, I had some concerns about the present findings both in terms of their theoretical significance and their effectiveness at validating the task paradigm. Unless these concerns can be addressed through adequate revisions, I'm not sure the present findings substantially advance our understanding of selective attention in memory.

We appreciate the reviewer's acknowledgement of the timeliness of the question.

We hope our responses below will clarify the primary goals of this study. Here, we aim to present a first proof-of-principle set of experiments to establish a new approach to a longstanding question; how selective attention operates on long-term memory content. With the vast majority of internal attention investigations operating solely on working memory content, we sought to design and test a methodology that utilized many of the same features well-established in the working memory literature. We take our share of the blame for not having made the background material, motivation, and rationale sufficiently clear for readers. We have introduced major changes to the revised manuscript, and we hope that the concerns raised by the reviewer are now all addressed.

– My main concern is that many of the findings reported here do not seem to tell us a clear story about the role of selective attention in LTM retrieval. This jeopardizes both the theoretical novelty of the findings and the practical value of the task paradigm for isolating attentional selection in LTM. Here are some examples:

- The cueing effects on RT seem fully explained by the number of perceptual comparisons performed at test. When the cue is invalid, participants first perform two perceptual comparisons using the cued target before switching to the un-cued target and repeating this process (3-4 comparisons). When the cue is valid, there is no initial sequence of rejecting the probes because the cued target is always present in the test array (1-2 comparisons). The neutral condition then falls right in the middle because the observer searches for the present target first on half of the trials on average (2-3 comparisons). If this is a fair interpretation of the results, it would mean that cueing effects on RT are contained entirely within the perceptual comparison stage at test and would be expected to occur in a WM paradigm as well.

The logic followed in our task design is the same as the extensively used and validated logic of internal selective attention tasks in working memory. The probe arrays are consistent across all experiments. They contain a target item previously associated with the scene and second item not part of the scene (new item in Experiment 1a or foil in Experiments 1b and 2). Spatially predictive cues guide the selection and prioritization of contents and facilitate responding at the probe stage. In the case of valid cues, participants will have selected the relevant object before probe onset, and this is ready for use for the 2AFC response. When the cue is invalid, they will have selected the other object, which they need to override and select the other scene-associated object. This leads to costs. When the cue is neutral, neither object is systematically prioritized, so performance is intermediate.

Now that the task designs provide clear benefits and costs of selective attention for retrieval, we can examine this further with neural methods. An account of the results that supposes that all of the effects occur at the time of the response probe is unlikely. This might explain differences in response times, but we also observe effects in accuracy.

However, more generally, we agree that the precise mechanism underlying the observed effects remains to be determined. In the current manuscript, we intentionally refrain from making strong mechanistic claims, as we believe that further studies, including neuroimaging ones, are required to reveal the neural mechanisms driving the behavioral benefits and costs.

- The authors claim that the cueing benefits on RT in the short interval condition of E2 indicate that attention selected the cued target directly from LTM rather than waiting until both targets had been retrieved back into WM to initiate selection. The data seems to directly contradict such a conclusion. In Figure 4, there is a clear main effect of interval on RT where both the valid and neutral cues produced slower RTs in the short interval condition than the long interval condition. This is exactly what we would expect to happen if observers needed to wait until the targets were retrieved back into

> WM to apply attentional selection. Note also that the RTs in the long interval condition in E2 were similar to—if not slower than—the RTs in E1B, which suggests that presenting the cue before the scene did not facilitate any processing that occurred before or during scene onset. If this is also a fair interpretation of the results, then the findings of E2 merely replicate cueing effects in WM that have already been shown many times over.

The logic behind our predictions for Experiment 2 was not sufficiently clear. We address this point (above) in our reply to common concerns raised by the reviewers.

In the revised manuscript, we have now spelled this logic out more clearly. The reasoning concerns whether the attentional benefit (in RT or accuracy) changes with the time available from retrieval onset until the response probe. If attention could only start to operate after all contents are in working memory, the benefits would be protracted (slower RTs) and less established (worse accuracy) when the interval is very short. If this was the case, we would have observed an interaction effect between the cue (informative vs. neutral) and interval (short vs. long) factors.

The reviewer is correct that we observed a main effect of interval, with slower RTs in the short compared to the long interval. However, critically, we did not observe an **interaction** between the interval and cue type. In other words, the size of the attentional orienting effect (valid vs. neutral) did not differ as a function of interval. This suggests that the attentional manipulation operated similarly under both time constraints.

The main RT effect of interval itself was not unexpected. A large body of literature (e.g., Woodrow, 1914; Los, 2010; Luce, 1991; Niemi and Naatanen, 1981) shows that short delays between a stimulus and a response lead to generally slower responses. This is commonly attributed to factors such as reduced motor readiness or less time for response preparation. The critical test in this context is whether the cueing effect changes across intervals, which it did not.

We are unsure whether we fully followed the point about the RTs in Experiment 2 and Experiment 1b. The retrieval conditions differ in the two cases. In Experiment 1b, participants had 2000 ms before the test appeared (1000 ms until the cue and another 1000 ms until the response probe). In the slow condition of Experiment 2, they had 1000 ms in total. In both cases, the retrieval period is relatively long and would provide enough time for retrieval. The important difference is between the RTs and accuracies obtained in the different cueing conditions.

Regarding the reviewer's final point, while our results may ultimately appear as if they replicate cueing effects reported by working memory studies, we still consider this a notable finding, as here we do not use a classical working memory design. In a typical working memory task, participants first see an object array and the attentional

manipulation happens after a short delay period. Here, attention does not act on content encoded seconds ago and still lingering within perceptual systems, but on information retrieved from long-term memory. What the reviewer may be implying is that attentional modulation happens in working memory. We believe this is a testable assumption, based on the standard view that long-term memory retrieval happens through working memory. Yet this topic remains highly debated (Fukuda & Woodman, 2017; Liu et al. 2022; Mugno & Vickery, 2025).

By using designs such as the one we introduce, and by coupling behavioral and neural methods, we hope in the future to test experimentally some of these theoretical pillars of the field. Further direct manipulations of working memory and long-term memory parameters within the current type of design will also help reveal the various steps, and the putative involvement of working memory, in long-term memory retrieval. What we offer is a first important step in this exciting direction.

- The cueing effect on accuracy was certainly intriguing, but the authors do not seem to provide a clear explanation for why this effect happened. For example, why did a cueing benefit on accuracy only occur in E2 and not E1B? Was it related to the timing of the spatial cue? The validity of the spatial cue? Was there a particular reason why the cue was rendered 100% valid only in E2?

We observed spatial cueing validity effects on accuracy in both Experiments 1B and 2. In Experiment 2, this took the form of a benefit, with higher accuracy in the valid versus neutral condition. In Experiment 1B, performance was higher for valid than neutral cues, but only the costs reached significance (invalid vs. neutral comparison). In both cases, the accuracy effects were complemented by attentional benefits in response times.

It is possible, as the reviewer suggests, that the timing of the cues influenced the ability to detect accuracy benefits. In Experiment 1b, participants had ample time between the scene and cue onset to retrieve the target, yielding high performance overall. In Experiment 2, the cues may have done more work during the retrieval process itself. Understanding how the dynamics of spatial attention and retrieval processes interact is certainly something our design renders tractable in future studies.

As for the last question, having established in Experiment 1 the feasibility of testing the attentional benefits for LTM retrieval, we began to examine the timing of the effects in Experiment 2. As per our pre-registration, we focused only on the more stringent comparison (attentional benefits: valid vs. neutral comparison). This was necessary to maintain a sufficient number of trials in each condition while not increasing too much the number of associations participants had to learn. Additionally, having two interval conditions (long vs. short) diminished the number of trials available for the different cueing conditions.

We have now explained the rationale for dropping the invalid condition in the methods of the revised text:

"Experiment 2 did not include invalid cues. The decision to focus only the valid vs. neutral contrast was based on the significant attentional benefits in Experiment 1b. The motivation was to maintain a sufficient number of trials per condition after introducing the additional retrieval-interval manipulation." (Page 11)

– At a higher level, I felt that the clarity of the narrative could be improved in the Introduction and Discussion.
    - o The Introduction opens with the claim that knowledge about selective attention in LTM is "scant" before then proceeding to outline a large body of literature that never seems to build toward a coherent research question or thesis. When I finished the introduction, I wasn't sure what was going to be tested or shown beyond "benefits of orienting selective attention in long-term memory".

Thank you for this helpful comment, which helps us make the main point of our study clearer. Our aim is to establish an experimental paradigm for investigating how selective attention operates in long-term memory. Our tasks enabled us to demonstrate directly selective attention benefits and costs guided flexibly and, on a trial-by-trial basis. This is a significant departure from the many convergent lines of work that demonstrate that attention is at work in long-term memory, without explaining how. We believe our task designs open doors for addressing many questions, some of which are longstanding and some of which are new.

Because we believe our work adds an important next step to the rich literature, we wanted to make sure to acknowledge the important and relevant foundation on which we are building. We may have been over-diligent. Based on the reviewer's comments, we extensively reworked the Introduction to explain and highlight the novelty of the work. In the revision, the description of prior work maintains the main points but is condensed.

- o The Discussion describes a plethora of future directions that are related to attentional selection in LTM, but many of these directions do not seem uniquely realized or afforded by the task that the authors are proposing. For example, the authors spend a good deal of time highlighting how their learning task allows researchers to manipulate the age and similarity between LTM representations. Those types of manipulations are already quite common in LTM research, and it was not clear to me how the present paradigm might be superior to those previous approaches.

We agree that we went overboard with this and have reduced the text substantially.

Regarding the reviewer's second point, our intent was not to imply that these were entirely new ways to manipulate long-term memory retrieval. Instead, we hope the current study will enable us and others to investigate how selective attention operates during retrieval

using some of the well-established methodology aspects commonly used in working memory studies. Specifically, we hope to be able to examine how attention interacts with well-established manipulations that change the strength, accessibility, or competition of memory contents. We have clarified this point in the Discussion.

"Furthermore, it is possible to test how the attributes of the long-term memories interact with attention functions. For example, the degree of item similarity impacts their competition during encoding and retrieval (Hutchinson et al., 2016; Kuhl et al., 2011; Wimber et al., 2015). By manipulating different types of item similarity (e.g., spatial proximity or sensory similarity), it will be interesting to test whether the strength of attention effects depends on the degree of competition, as it does in sensory (Kastner & Ungerleider, 2001; Mitchell et al., 2009) and working-memory (Ahmad et al., 2017; Shapiro & Miller, 2011) domains. In addition to the qualities of the associations, their strength and age may also matter. By using separate Learning tasks, the experimenter gains control over these parameters. (page 20)

—

"Our experimental approach thus paves the way for addressing many questions about long-term memory retrieval, some longstanding and others new. Fruitful topics include the types and time courses of selective attention functions; their dependence on intermediary working memory representations; and their interaction with memory quality, strength, and competition. We suspect these tools will yield interesting insights into this rich and foundational intersection between memory and attention." (page 20).

– I may have missed it, but can the authors provide some information about number of trials that were excluded for exceeding the 3000ms response window at test? Did these numbers differ at all between conditions or experiments?

Thank you for pointing out this missing information. Only a very small number of trials were excluded for participants not responding within the 3000-ms time window during the Selective Retrieval Task, and these did not differ between conditions. We have now included the information to the Results section.

- Experiment 1a: M = 1.05 trials; SD = 2.54.
- Experiment 1b: M = 1.24 trials; SD = 3.04.
- Experiment 2: M = 1.30 trials; SD = 2.54.

4. Reviewer #3 (Remarks to the Author):

Summary: This three-experiment study aims to develop an experimental paradigm that supports the direct study of attention orienting in long-term memory (LTM). Participants in all three experiments first carried out a learning task in which they learned the pairings of scene contexts with two objects (each). Once having learned these pairings, participants' memories were probed in a task in using valid, invalid, and neutral retrocues in Experiments 1 & 2 and valid and neutral pre-cues in Experiment 3. Participants made 2AFC responses in each task selecting which of two items in a probe array is the target item. The results in general show a benefit of valid cueing and a cost of invalid cueing across the tasks. The authors conclude that these results support the use of this paradigm for further and deeper investigation of the mechanisms supporting selective attention from LTM.

Main comments:

In all, I find the manuscript to be methodologically sound. The use of scene–object associations and controlled cueing manipulations can provide a useful framework for examining how predictive information biases memory retrieval.

Thank you.

However, the theoretical claims—particularly those concerning selective attentional orienting within LTM—appear to extend beyond what the current behavioral evidence can support. So, while I believe the experiments provide a solid demonstration that predictive cues bias retrieval from LTM, the current data more strongly support an account based on guided or selective retrieval than one invoking selective attentional orienting within LTM representations.

Specifically, the manuscript frequently uses the term "selective attention" to describe the effects of informative cues on retrieval from LTM. Although this terminology is well-established in the context of working memory, it's unclear whether that is what is being measured here. The observed cueing benefits are consistent with selective retrieval or expectancy-based preparation and may not necessarily reflect attentional selection operating over competing LTM representations (see comments below). I think that clarifying whether the intended claim concerns attentional selection per se or selective retrieval more broadly would strengthen the theoretical framing and interpretation of the results.

Thank you for these comments. This is an important point for us to explain and flesh out in the paper. The reviewer's takeaway is precisely the main point we are trying to make, that "predictive cues bias retrieval from LTM … an account based on guided or selective retrieval…".

In our theoretical framing selective attention comprises the functions that anticipate, select, and prioritize content to guide adaptive behavior according to goals and expectations (see Nobre and van Ede, 2018, 2023; Nobre and Stokes, 2019). These attention functions can work prospectively to modulate the processing of incoming sensory information (external attention) or retrospectively to modulate pre-existing memory contents (see Chun et al., 2011; Nobre & Stokes, 2019; van Ede & Nobre, 2023). In our view these attention functions are an integral part of selective retrieval. Our aim with designing these new tasks is to provide an experimental approach to investigate *how* selection and prioritization (i.e., attention) functions operate to guide retrieval.

We have defined more clearly what we mean by attention and how this relates to selective retrieval in the revised manuscript.

> *"In our framework, memories of different types and lifetimes are proposed to furnish experience-related traces. Selective attention is the set of functions that utilize these memory traces to anticipate, select, prioritize, and prepare contents to shape perception, cognition, and action (Van Ede & Nobre, 2023). In this framework, attention can work on contents originating in the sensory stream (external) or on pre-existing representations untethered to sensory signals, such as memory contents (internal)." (page 3)*
>
> —
>
> *"That" attention plays a core role in long-term memory is hardly questioned. Some even propose that selective retrieval is equivalent to internal attention in long-term memory (Chun et al., 2011; Long et al., 2018)." (page 3).*
>
> —
>
> *"The current study helps promote the view that attention functions are an integral part of selective retrieval (Chun et al., 2011; Long et al., 2018). The findings complement previous work done in this area (e.g., Anderson et al., 1994; Clément & Tallon-Baudry, 2025; Hutchinson et al., 2016; Kuhl et al., 2013; Rugg & Wilding, 2000; Sabo et al., 2024; Sabo & Schneider, 2022; Waldhauser et al., 2012, 2016; Wimber et al., 2015)." (page 18).*

Across the three experiments, the general finding is that valid cues tend to facilitate performance relative to neutral cues, and (when included) invalid cues incur costs. Although this pattern is reliable, it may also be a canonical consequence of predictive cueing as has been well-documented in both working memory and retrieval cueing literatures. Such effects can arise from strategic prioritization, expectancy-driven preparation, or decision-level bias (e.g., Souza & Oberauer, 2014; Posner, 1980; Günseli et al., 2015). Therefore, the results do not adjudicate among these possibilities and point specifically to selective attention operating over LTM representations.

We agree that the pattern of valid-cue benefits and invalid-cue costs observed across the three experiments could, in principle, arise from multiple mechanisms. Indeed, whereas many researchers have sought to single out the specific mechanism that accounts for the effects of selective attention in working memory, we have specifically argued demonstrated in previous work (see van Ede and Nobre, 2021, 2023; Nobre and van Ede 2023) that the brain has many ways to enhance performance through internal attention. This includes enhancing content, facilitating access, reconfiguring neural code, protecting from decay, protecting from interference, reducing competition, recruiting action planning, etc. (see van Ede and Nobre, 2021, 2023; Nobre and van Ede 2023).

Our goal was to establish an experimental framework for investigating how attentional orienting shapes long-term memory retrieval. We believe that further behavioral and neuroimaging studies will be needed to examine the processes underlying these effects more thoroughly. Additionally, future work will have to determine the various attention mechanisms for modulating long-term-memory retrieval depending on memory-related and task-related factors.

An additional factor contributing to ambiguity in interpretation is the structure of the probe arrays. Participants are required to select the target object from a probe array also containing foil that is either novel or associated with a different scene. Even on invalidly cued trials, the probe array contains only a single object from the scene (the target) alongside an irrelevant foil, such that participants are not required to resolve competition between multiple concurrently relevant representations from the same long-term memory context. Consequently, performance can be optimized by prioritizing a single expected item rather than by selectively enhancing one representation while suppressing another. Although invalid trials demonstrate that participants can retrieve uncued information when the cue misleads them, this outcome reflects flexibility of retrieval following misleading cues rather than evidence for attentional selection operating simultaneously over multiple LTM representations.

We do not disagree with this. We believe that the spatial cues in our tasks act by selecting and prioritizing one of the objects associated with the scene. The invalidity costs show some degree of relative de-prioritization of the other object, but this could be a flexible and reversible process. Future studies manipulating spatial attention in long-term memory could specifically test for the suppression of the non-attended item, in an analogous way to manipulations introduced in retrieval-induced forgetting paradigms. Subsequent memory tasks could compare retrieval performance of unprobed objects or object-scene associations when spatial cues pointed to another item or when neutral cues did not prioritize either object.

The probabilistic cue validity manipulation in Experiments 1 and 2 (80% valid, 20% invalid) further encourages strategic retrieval preparation. Preparing for the cued object as the most

likely response naturally produces both benefits on valid trials and costs on invalid trials, even in the absence of attentional modulation of memory representations. Therefore, I think the manuscript would benefit from more explicitly clarifying how such expectancy-based or decision-level mechanisms are distinguished from attentional orienting within LTM.

Hopefully, our explanations based on the reviewer's comments above have helped clarify our theoretical framing. The revised text makes the stance clear and states that we believe attention functions are intrinsic to selective retrieval. Using our approach, we wish to investigate how these attentional functions operate and how they change retrieval outcomes.

We deliberately refrain from providing mechanistic interpretations of the behavioral benefits and costs. Once the phenomenon of selective attention during retrieval is established (our current goal), we foresee many lines of behavioral and neural investigations that can reveal the mechanisms by which benefits and costs arise. We note, however, that the response measure we chose (2AFC) helps overcome issues related to decision biases and criterion shifts. It provides a sensitive measure of the presence of memory traces by providing both a memory target and nontarget option on every trial and requiring the participant to choose which of them elicits a stronger memory signal.

Experiment 3, while informative in demonstrating cue-driven facilitation, removes several features that could reveal selective attention. The absence of invalid cues eliminates reorienting demands and competitive selection, limiting the experiment's ability to speak to selective attention mechanisms. Although the cue-first design is interpreted as demonstrating attentional facilitation of LTM retrieval independent of working memory, it remains possible that the target object is loaded into working memory once the scene appears. The observation that response times are slightly faster following the longer delay is consistent with participants using this interval to pre-activate the relevant representation, suggesting that performance benefits may still be mediated via working memory maintenance rather than solely reflecting attentional selection over long-term memory.

It is true that we restricted the attentional cueing conditions of Experiment 2 to valid and neutral. This was done to maintain a high trial number in all conditions while also keeping the number of scenes participants had to learn manageable. (This is now explained in the methods section of the revised manuscript.) Although the choice restricted us in examining attention-related costs, it still enabled us to measure the consequences of valid spatial cueing (valid vs. neutral contrast). The approach is standard practice in many attention studies, including internal attention studies in working memory (e.g., van Ede et al. 2020).

We interpret the findings of our third experiment (Experiment 2) as suggestive that spatial attention can influence retrieval from early stages. We also acknowledge that further work will be required to vindicate our hunch.

Our logic may not have been sufficiently spelled out in the original paper, so we have included a new section explaining the thinking. Please see our reply to common points raised by the reviewers.

As the reviewer notes, response times are faster when participants have longer retrieval intervals. However, slower responses after a very short foreperiod (300 ms) are standard in most types of speeded response tasks (not just in memory). As we note to Reviewer 2, a large body of literature (e.g., Woodrow, 1914; Los, 2010; Luce, 1991; Niemi and Naatanen, 1981) shows that short delays between a stimulus and a response often lead to generally slower responses. This is commonly attributed to factors such as reduced motor readiness or less time for response preparation. The critical test in this context is whether the cueing effect changes across intervals, which it did not. Of note, if we consider the retrieval times in relation to the appearance of the scene, then participants respond much earlier in the short-interval trials.

In the revised text, we explain the logic of the manipulation and acknowledge that, though suggestive, our results are inconclusive as to whether retrieval can occur entirely without working memory mediation. (Please see reply to common concerns by the reviewers.)

Considering other performance patterns: Performance appears modestly worse in Experiments 2 and 3 relative to Experiment 1, with slower response times and slightly reduced accuracy, though no formal cross-experiment comparisons are reported. These patterns suggest increased task difficulty or inter-participant strategic variability without clear evidence of enhanced attentional selectivity. Moreover, accuracy does not vary across delay lengths in Experiment 3, consistent with effective maintenance of the relevant representation, while the slight speeding of response times at longer delays may reflect more efficient pre-activation rather than decay-resistant attention operating directly within LTM.

Thank you for the observations. As the reviewer notes, Experiment 1a was easier than Experiments 1b and 2. This was by design, as specified in our preregistration. In Experiment 1a, responses reflected a simpler old/new decision and could be based on familiarity or recollection. Experiments 1b and 2 required retrieval of the specific scene-object association to distinguish the target from the foil. Our choice to change the required memory response had two motivations. (1) Behavioral performance in Experiment 1a was close to ceiling, which complicated measuring attentional effects on accuracy. (2) Retrieval of the specific scene-object association is a more relevant indication of selective prioritization of content within a memory context, which was our direct interest.

Because of the reasons mentioned above, a comparison of effects across the three experiments was not part of our pre-registration. Nevertheless, based on the reviewer's suggestion, we have now added an exploratory between-experiment ANOVA with the factors cue (valid, invalid, neutral) and experiment (1a vs. 1b) (for the descriptive statistics, see Table 1). We restricted the ANOVA to these two experiments because they are equivalent in

conditions and trial numbers, with only the nature of the memory test differing. Results can be found on pages 15-16 of the Results section.

> *"Accuracy results revealed a main effect of cue condition, F(2, 296) = 9.72, p < .001, BF10 = 5425.22, a main effect of experiment, F(1, 148) = 48.40, p < .001, BF10 = 6.45×107, and a cue condition x experiment interaction, F(2, 296) = 9.34, p < .001, BF10 = 7.24×1013. Analyses of response times yielded a main effect of cue condition, F(2, 296) = 42.23, p < .001, BF10 = 9.64×1016, a main effect of experiment, F(1, 148) = 33.74, p < .001, BF10 = 149191.01, but no cue condition x experiment interaction, F(2, 296) = 1.30, p = .27, BF10 = 0.68." (pp. 15-16).*

As the reviewer surmised, the ANOVA indicated that performance was better in Experiment 1a than 1b, with higher accuracies and faster RTs. While the high accuracy in Experiment 1a showed no modulation by attention, it was significantly impacted in Experiment 1b. Response times effects were comparable across experiments.

Table 1

Descriptive statistics – accuracy (%)

condition	experiment	N	Mean	SD	SE
valid	1a	60	97.55	3.76	0.48
	1b	90	88.32	9.99	1.05
neutral	1a	60	96.74	4.69	0.60
	1b	90	87.28	10.86	1.14
invalid	1a	60	97.23	4.56	0.59
	1b	90	83.07	16.97	1.78

Descriptive statistics – response times (ms)

condition	experiment	N	Mean	SD	SE
valid	1a	60	782.33	176.09	22.73
	1b	90	980.73	237.66	25.05
neutral	1a	60	797.69	176.03	22.72
	1b	90	1013.40	245.08	25.83
invalid	1a	60	836.47	176.02	22.72
	1b	90	1054.58	262.17	27.63

Minor comments:

i. The title in the manuscript file differs from what is in the online submission.

We apologize for this inconsistency. The title we are proposing is “Orienting selective attention within long-term contextual memories”.

ii. Were there any performance differences between Day 1 and Day 2? If learning carried over, then perhaps Day 2 effects would be stronger and more LTM-based?

The experiments conducted on the two days used entirely different sets of scenes and objects, with no overlap between days. Therefore, we do not expect learning of specific associations to carry over from Day 1 to Day 2. At most, one might expect general practice effects, in the sense that participants are more familiar with the task procedure on Day 2.

> *“Each session used entirely different sets of scenes and objects, with no overlap between days.”* (Procedure, page 8)

iii. For clarification purposes: were there only four object locations or were locations more jittered than that? And were object locations within the scenes always left and right (meaning one object was always on the left and one always on the right)?

In all three experiments, objects could appear in eight possible locations: four on the left side of fixation and four on the right side. On each trial, one object always appeared in one of the left-side locations and the other in one of the right-side locations. We have now added this information to the Methods section (see page 9).

> *“Within each scene, one object was on the left and the other on the right side. Objects could occupy one of four positions on each side. Object locations were: upper left (PsychoPy units: -0.325, 0.188 and -0.188, 0.325), lower left (PsychoPy units: -0.325, -0.188 and -0.188, -0.325), upper right (PsychoPy units: 0.325, 0.188 and 0.188, 0.325), and lower right (PsychoPy units: 0.325, -0.188 and 0.188, -0.325). The eight locations were counterbalanced across participants and equally distributed across objects and trials.”*

iv. The way that I understood the response ‘grading’, if the participant clicked on the correct quadrant, then the response was recorded as ‘correct’, otherwise, ‘incorrect’. But, I wonder if there was a more continuous measure for accuracy, specifically, how far the click was from the actual location or were they just told to click on one of four quadrants?

Responses were scored based on the selected quadrant. We chose this approach for pragmatic reasons, as the study was conducted online. This method provided a straightforward implementation. Moreover, the primary purpose of the Learning Task

feedback was to reinforce encoding rather than to obtain a fine-grained measure of spatial precision. In future laboratory-based studies, we plan to investigate memory precision using more sensitive measures.

v. Were there any performance differences for objects in the left part of the display versus the right? Or when the target probe was upper or lower? If participants used a particular and consistent scanning strategy, this could lead to behavioral differences according to these factors.

Thank you. This was worth checking. Item locations were fully counterbalanced across conditions. Within each trial, one object was presented on the left side of fixation and the other on the right. To examine whether performance differed depending on whether the target object had originally appeared on the left versus the right, we conducted paired-samples t-tests for each experiment. Accuracy did not differ between left and right target locations:

- Experiment 1a: $t(59) = -0.17$, $p = .86$, $BF_{01} = 6.98$.
- Experiment 1b: $t(89) = 1.68$, $p = .09$, $BF_{01} = 2.22$.
- Experiment 2 : $t(84) = -0.86$, $p = .39$, $BF_{01} = 5.82$.

We also tested whether performance differed depending on whether the correct probe in the 2AFC display appeared in the upper or lower position. This was fully counterbalanced across trials (50% top, 50% bottom). If participants had adopted a consistent scanning strategy (e.g., always checking the top item first), this should have resulted in systematic accuracy differences. However, accuracy did not differ between top and bottom correct-response trials, and Bayes factors supported the null hypothesis:

- Experiment 1a: $t(59) = -0.23$, $p = .81$, $BF_{01} = 6.90$.
- Experiment 1b: $t(89) = -1.15$, $p = .25$, $BF_{01} = 4.51$.
- Experiment 2 : $t(84) = 0.92$, $p = .36$, $BF_{01} = 5.54$.

vi. An alternative explanation that came to mind is that participants may maintain/activate representations of both objects associated with a scene and use a comparison/search process at probe to identify the correct target. The observed cueing benefits and costs could then arise from biases in search order or retrieval readiness rather than from selective attentional orienting operating over LTM representations. Is there anything in the data that could address this or rule this out?

The scenario described by the reviewer is theoretically possible. However, we believe that some aspects of the data make a pure search-order or retrieval readiness account unlikely. A comparison/search account would primarily predict differences in response times.

However, we also observe systematic effects on accuracy (i.e., benefits for valid cues and/or costs for invalid cues).

In the revised discussion, we suggest the following justifications for our preferred interpretation:

> *"We favor the former possibility, for two main reasons. In everyday situations, successful selective retrieval operates without us being presented with outcome options and having to decide among all possible alternatives. Furthermore, previous studies using brain imaging have also clearly highlighted goal-related modulation of memory representations during retrieval before the presentation of response arrays (e.g. Kuhl et al., 2012). However, we acknowledge that future studies incorporating neural measures into our designs will be better equipped to track the stages of modulation." (page 19)*

References

Chun, M. M., Golomb, J. D., & Turk-Browne, N. B. (2011). A taxonomy of external and internal attention. Annual review of psychology, 62(1), 73-101.

Fukuda, K., & Woodman, G. F. (2017). Visual working memory buffers information retrieved from visual long-term memory. Proceedings of the National Academy of Sciences, 114(20), 5306-5311.

Liu, B., Li, X., Theeuwes, J., & Wang, B. (2022). Long-term memory retrieval bypasses working memory. NeuroImage, 261, 119513.

Los, S. A. (2010). Foreperiod and sequential effects: Theory and data. In Attention and Time (pp. 289302). Oxford University Press. DOI: https://doi.org/10.1093/acprof:oso/9780199563456.003.0021

Luce, R. D. (1991). Response Times: Their Role in Inferring Elementary Mental Organization. Oxford University Press.

Mugno, M. K., & Vickery, T. J. (2025). Retrieval from long-term memory does not bypass working memory. Attention, Perception, & Psychophysics, 87(8), 2275-2286.

Niemi, P., & Naatanen, R. (1981). Foreperiod and Simple Reaction Time. Psychological Bulletin, 89(1), 133–162. DOI: https://doi.org/10.1037/0033-2909.89.1.133

Nobre, A. C., & Stokes, M. G. (2019). Premembering experience: A hierarchy of time-scales for proactive attention. Neuron, 104(1), 132-146.

Nobre, A. C., & van Ede, F. (2023). Attention in flux. Neuron, 111(7), 971-986.

van Ede, F., & Nobre, A. C. (2021). Toward a neurobiology of internal selective attention. Trends in neurosciences, 44(7), 513-515.

Van Ede, F., & Nobre, A. C. (2023). Turning attention inside out: How working memory serves behavior. Annual review of psychology, 74(1), 137-165.

Woodrow, H. (1914). The measurement of attention. Psychological Monographs, 17(5), 1–158. https://doi.org/10.1037/h0093087

We include the editor's and reviewers' comments in black font and provide our replies in light blue. To facilitate the reviewing process, we transcribed the core changes in the text in *italicized blue text.*

Replies to editors' comments:

The editors' clear guidance on the priorities for the revisions was extremely helpful.

(1) more clearly position the contribution of the present findings relative to prior work on attentional prioritization in working memory

We agree that the paper's distinctive contribution is more clearly conveyed by being explicit about what is — and what is not — extended beyond existing working-memory work. We have rewritten the closing paragraphs of the Introduction (pages 5-6) and the opening of the General Discussion (pages 19-20) to state directly that: (a) the novelty lies in showing that the well-developed toolkit of retrocueing cannot only be used to study contents coming from perception, but also content reactivated from long-term memory; (b) the present data establish that selective prioritization operates in this setting, with magnitudes broadly consistent with the working-memory literature; and (c) the present behavioral data do not, on their own, establish that the locus of modulation lies before working-memory reinstatement. We previously implied a stronger claim in places and have tempered the language accordingly. See revised Introduction (last paragraphs, pages 5-6) and the new "Interpretations" (pages 19-20) paragraph in the General Discussion.

(2) address alternative accounts based on post-retrieval comparison processes

We have added an explicit section in the General Discussion, in which we discuss the locus of the modulation (see pages 19-20) and discuss how attention might have occurred at different stages, including after working-memory reinstatement (i.e., post-retrieval stage). We acknowledge that this account can in principle explain both response-time and accuracy effects. Our previous reply had argued that the post-retrieval stages scenario could account for response-time but not accuracy effects. We now retract that distinction, which we are no longer confident is warranted. We are clear in the revised text that the present design cannot definitively settle at which stage attention operates, doing so will require neural or time-resolved measures that index modulation prior to probe onset.

(3) clarify the assumptions underlying the interpretation of Experiment 2, especially with respect to claims about mediation by working memory.

Our previous framing rested on the implicit assumption that on the short-interval trials, prioritization in working memory would not be completed before probe onset, which would in turn attenuate the cueing benefit. We agree with Reviewer 2 that this assumption

might not have been fully justified and that an equally tenable model is that retrieval-plus-prioritization completes after probe onset on short-interval trials, producing a comparable cueing benefit in both delay conditions. The revised manuscript now states this explicitly (pages 5-6 and 19-20). We have kept the interval manipulation in the paper because we consider the finding that cueing benefits survive aggressive shortening of the retrieval window informative for future work. But we have removed the claim that the absence of an interaction is diagnostic of an early, pre-working-memory locus of modulation. We offer our preferred interpretation as only one option out of multiple possible alternative interpretations (Discussion 19-20). We have also revised the relevant section in the Introduction (pages 6-7).

Replies to reviewers' comments:

Reviewer #1:

The authors have responded thoroughly to my concerns on their previous submission. I believe this version of the manuscript is much improved, and I have no further comments.

We thank Reviewer 1 for their careful engagement. We are pleased they judged the manuscript to be improved and to address their concerns.

Reviewer #2:

The authors have made significant revisions to their manuscript which have greatly improved the clarity of their arguments and tightened the theoretical focus. However, I'm still not strongly convinced that this study is making a substantive contribution that moves beyond what we've already gained from studies of attentional priority in WM. The focus on LTM is well-motivated, but the results only appear to replicate what has already been shown in WM studies now using retrieved LTMs instead of recent percepts. The authors do attempt to produce a theoretical advance that is unique to LTM in E2, but it seems that the authors and I both agree that the evidence is not compelling enough to truly challenge existing theory.

Below, I outline my concerns in detail to have them on the record as part of my review. However, I'm happy to defer my recommendation to the discretion of the editor and the consensus of the other peer reviewers. If these folks feel that the findings reported in this current version of the manuscript are substantive enough to warrant publication, then the paper should be moved forward.

We are grateful to Reviewer 2 for their continued, substantive engagement with the manuscript. We respect their views and appreciate both their candor and flexibility.

In our revision, we further contextualize our study, flesh out premises, and motivate why we believe we offer a first step into what is likely to be a fruitful field of investigation. Whether the

mechanisms of internal attention differ depending on the origin of contents, either from the sensory stream or from latent long-term memories, is an important and empirical question. What we offer is a well-controlled approach for engaging directly with these types of questions.

1. I raised the possibility that the cueing effects observed could be explained by the number of perceptual comparisons performed at test. My original intent was not to challenge the claim that people prioritize one of the two items prior to the report (though I can see how this was not clear from my original comments). On the contrary, prioritizing the cued item prior to the report is what ensures that it is used for comparison first. Instead, my point was that, if the effect of priority on accuracy and RT can be reduced to the number of comparisons performed at test—or any other process that occurs *after reinstatement into WM*—then the cueing effects reported here do not demonstrate an influence by attention that was *unique to LTM* and differs in a *meaningful way* from effects already shown in WM studies.

So, can these results be explained by differences in the number of comparisons? The authors claim that the number of comparisons made at test can account for cueing effects on RT but not on accuracy, but no rationale is provided for the latter claim. On the contrary, there is ample evidence showing that memory probes induce interference that is greater in de-prioritized items than prioritized items (see, e.g., Makovski et al., 2008 JEPLMC; Saito et al., 2025 JEP:G). Therefore, it seems perfectly plausible that performing 1-2 additional probe comparisons (1) slows reports and (2) makes them slightly less accurate.

We thank the reviewer for clarifying their original point and for noting that prioritization of the cued item before the probe is a shared premise between our account and theirs. We had, indeed, interpreted the original comment differently.

We accept the possibility that the cueing benefits might be explained by processes that occur after reinstatement of long-term memory contents into working memory (e.g., serial comparison order; probe-induced interference that disproportionately degrades de-prioritized items). We are grateful for the pointers to Makovski et al. (2008) and Saito et al. (2025). The demonstration that probe-induced interference is greater for de-prioritized items is directly relevant and undercuts a distinction we had previously drawn between response-time and accuracy effects. In our prior reply, we had suggested that comparison-count effects could account for response-time but not accuracy differences. However, the reviewer has convinced us, and we retract the argument we originally made about the difficulty of the account in explaining the accuracy effects.

Thus, the reviewer offers a legitimate live alternative that our behavioral data cannot rule out.

Even in this case, there are two possible scenarios. (1) As suggested by the reviewer, the effects we observe echo those routinely demonstrated in retrocueing studies of contents maintained in working memory after sensory encoding. (2) An alternative which intrigues us is the possibility that contents placed in working memory may be in different states or have different

properties depending on whether they have recently been encoded into working memory from the sensory stream or retrieved from long-term memory. For example, the degree of representational fidelity and competition with other related traces may differ. Accordingly, although the studies highlighted by the reviewer (Makovski et al., 2008; Saito et al., 2025) demonstrate these effects for recently encoded working-memory contents, an open question is whether the same mechanisms apply when working-memory representations originate from long-term memory retrieval.

We believe it will be extremely interesting to understand if internal attention during long-term memory retrieval: (1) is necessarily mediated by working memory and is just the same as that operating on working memory content with sensory origins, (2) is mediated by working memory but has different properties because the reinstated working-memory representations differ qualitatively from the freshly encoded representations, or (3) operates directly on long-term memory representations independently of working-memory reinstatement. What we provide is a way into these fundamental questions and some early, promising findings.

The following new text was included in the revised manuscript to address these issues:

> *"… Thus, our aim is to extend the experimental toolkit of internal-attention retrocueing, developed for working-memory contents, to contents reactivated from long-term memory. Exploring internal attention to contents originating from long-term memory should open doors to important and longstanding questions. For example, if cueing significantly enhances selective long-term-memory retrieval, future studies will be able to test directly the role that working memory plays in the process. Three scenarios are possible: (1) Selective long-term-memory retrieval requires the initial reinstatement into working memory with internal attention operating in the same way as it does on working memory contents encoded from the sensory stream. (2) Selective long-term-memory retrieval requires the initial reinstatement into a working memory buffer. However, contents reinstated from long-term memory may differ in their qualities or properties compared to items freshly encoded into working memory from the sensory stream, so that the consequences of internal attention may also differ systematically. (3) Finally, internal attention may operate directly on long-term memory representations independently of working-memory reinstatement, modulating retrieval in distinct ways. " (Introduction, page 5)*

> *"In principle, the selection and prioritization of memory contents might operate at three different stages. (1) Attention functions may start modulating long-term memory contents very early on, before the representations are transferred to working memory. This is plausible, given the existence of functional anatomical connections between long-term memory control regions (e.g., in medial temporal areas) and visual regions required for retrieving the sensory qualities of the associated contents. In this scenario, selection and prioritization of long-term-memory contents may occur before they reach an active state in working memory, recruiting prefrontal and parietal areas. (2)*

Alternatively, long-term memory retrieval may necessarily involve reinstatement and mediation via working memory. Selection and prioritization may occur only after the long-term memory contents have been transferred to working memory. (3) A final possibility is that attention functions do not impact the retrieval process itself, but instead operate later, at the response stage. In this case, attentional processes may help guide the decision of which retrieved content is relevant or correct. These three possibilities are not mutually exclusive, and there could be hybrid or other variations." (Introduction, page 6)

"Our findings raise the intriguing possibility that selection and prioritization of long-term memory contents may start very early in the retrieval process, ***although alternative explanations remain open****." (Discussion, page 19)*

2. I and another reviewer raised the concern that the findings in E2 do not challenge the assumption of mediation by WM in LTM retrieval. We based this concern on the main effect of interval in RT, which showed that reports were delayed in the short interval condition compared to the long interval condition across cues, which would be expected if observers had to wait until retrieval into WM was complete to prioritize memoranda. The authors claim that mediation by WM should have produced an interaction between interval and cue validity, where cueing benefits in RT and accuracy are weaker in the short interval condition than the long interval condition. Much like their claim described in comment #1, the rationale for this claim is never clearly articulated. Based on what was provided, the authors seem to be saying that retrieval is slow, so when the SOA is extremely short, people should not have enough time to recall both items into WM and prioritize one of them before the report probes onset. As a result, on some trials prioritization never actually happens, which should reduce the size of the cueing benefit when averaging across trials. To make sense of the main effect of interval on RT, the authors suggest that the delay could be explained by differences in motor readiness or response preparation.

Critically, the only way that this interaction can be used as grounds to test for mediation by WM is if all of the assumptions above are true, but no evidence is provided to substantiate any of them. As such, it remains equally tenable that items were retrieved into WM and prioritized even after the report probes onset, which produced a comparable cueing benefit in the short condition that was slightly delayed relative to the long condition. Of course, the authors are welcome to continue taking a side on this issue in their General Discussion, but they should not unfairly ground the issue on an interaction that may not actually be diagnostic of mediation by WM.

We agree with Reviewer 2 on this point and have made the corresponding changes in the manuscript. Our framing implicitly assumed that the aggressive shortening of the retrieval interval in Experiment 2 would provide insufficient time for full reinstatement into working

memory, followed by selection and prioritization, before probe onset and response completion. This would result in incomplete or absent prioritization in some trials and a smaller cueing effect overall. This is still our preferred putative explanation, but we understand that the case is not airtight. The possibility that Reviewer 2 articulates is also plausible. Future studies using additional behavioral (e.g., gaze biases) or neural (e.g., EEG) measures will help adjudicate between the possibilities.

The revised text presents the retrieval interval finding as informative in a more modest sense. Specifically, the paper now highlights that cueing benefits are robust under substantial time pressure, which constrains the time course over which the relevant modulation must be in place. Yet, it does not, on its own, prove that the modulation happens before working-memory reinstatement. We retain a demarcated theoretical paragraph in the Discussion in which we lay out our preferred interpretation and our reasoning for favoring it (consistent with reviewer 2's invitation to do so). However, we also offer the alternative explanation and note that future investigations are required to test between the different theoretical positions.

> *"… if selective attention only acts after the long-term memory content is in working memory (scenario 2), its benefits should be intact with a long retrieval interval but would be compromised with a short retrieval interval. This would occur because, in the short interval condition, reinstatement would not be complete when the probe appears. However, we cannot exclude the possibility that the processes of memory reinstatement and attentional prioritization continue to unfold after the probe presentation." (Introduction, page 7)*

> *"… Although the results do not provide definitive explanations, they leave open all possibilities, including one in which long-term memory retrieval is highly flexible and modulated by goals and expectations from the very first steps. We favor this highly flexible view of long-term memory retrieval, but future studies with time-resolved behavioral or neural measures will be required to arbitrate among the competing alternatives. It is also entirely possible that attention-related modulation occurs at more than one processing stage." (Discussion, pages 19-20)*

Reviewer #3:

The authors satisfactorily addressed my previous comments. The revised narrative, in particular, improves the flow of the manuscripts and the framing of the study. The other clarifications in response to all of the comments raised by the reviewers makes clear the aims of the study and properly situates the results in the literature.

We thank Reviewer 3 for their support and detailed constructive comments through the review process. The points were extremely helpful in guiding us to clarify and articulate our motivations and interpretations.
