# Supplementary material for: Orienting selective attention within long-term contextual memories
Source: Commun Psychol. 2026 Jul 16;4:145. doi: 10.1038/s44271-026-00505-9 (PMC13690599; doi:10.1038/s44271-026-00505-9)
Supplement: Supplementary file 2 [file 44271_2026_505_MOESM2_ESM.pdf]

Orienting selective attention within long-term contextual memories

Supplementary material

Table S1a. *Descriptive statistics for encoding blocks of the Learning Task in Learning Experiment 1a.*

Experiment	Parameter	Block	Test Order	N	Mean	Standard error of the mean
Experiment1a	Accuracy (%)	block1	first	60	98.40	0.30
			second	60	99.30	0.20
		block2	first	60	98.20	0.30
			second	60	99.00	0.20
		block4	first	60	98.40	0.30
			second	60	99.20	0.20
		block5	first	60	98.30	0.30
			second	60	99.20	0.20
	RT (ms)	block1	first	60	941.01	32.36
			second	60	523.29	29.71
		block2	first	60	916.26	27.35
			second	60	493.39	30.17
		block4	first	60	899.68	27.01
			second	60	475.26	31.59
		block5	first	60	895.07	28.28
			second	60	457.72	31.06

Table S1b. *Descriptive statistics for the testing block of the Learning Task in Learning Experiment 1a.*

Experiment	Parameter	Block	Test Order	N	Mean	Standard error of the mean
Experiment1a	Accuracy (%)	block3	first	60	76.70	2.10
			second	60	84.90	1.70
	RT (ms)	block3	first	60	1246.31	35.41
			second	60	845.90	33.02

Table S1c. Repeated Measures Analyses of Variance (rm-ANOVAs) on accuracies and response times from the encoding blocks of the Learning task in Experiment 1a. Significant effects are underlined.

Effects	df1	df2	F	*p*	η^2_p & 95% CI	BF_{10}	interpretation
**Experiment 1a – accuracy**							
Block	2.61	177	0.86^{corr}	0.450^{corr}[1]	0.01 [0, 0.05]	0.05	substantial evidence for null model
Test order	1	59	43.23	< .001	0.42 [0.24, 0.57]	750947.65	very strong evidence for the main effect of test order
Block*Test order	3	177	0.29	0.82	0.005 [0, 0.02]	0.006	strong evidence for null model
**Experiment 1a – response times**							
Block	1.82	107	12.16^{corr}	< .001^{corr}[2]	0.17 [0.06, 0.29]	49279.55	very strong evidence for the main effect of block compared to a null model
Test order	1	59	712.06	< .001	0.92 [0.89, 0.95]	2.96× 10^{+31}	very strong evidence for the main effect of test order comparted to a null model
Block*Test Order	2.17	128	0.62^{corr}	0.55^{corr}[3]	0.01 [0, 0.06]	0.18	substantial evidence for the null model

Table S1d. One-sample t-test against chance (0.25) on accuracy – test block of the Learning task in Experiment 1a.

	t	df	*p*	Cohen's *d* & 95% CI
**Experiment 1a**				
First object location test	24.76	59	< .001	3.19 [2.56, 3.82]
Second object location test	34.31	59	< .001	4.43 [3.59, 5.25]

[1] Since the sphericity assumption was violated, Greenhouse-Geisser corrected results are reported: $\chi^2(5)=17.18$, $p=.004$, $\varepsilon=0.87$.
[2] Since the sphericity assumption was violated, Greenhouse-Geisser corrected results are reported: $\chi^2(5)=53.92$, $p<.001$, $\varepsilon=0.60$.
[3] Since the sphericity assumption was violated, Greenhouse-Geisser corrected results are reported: $\chi^2(5)=31.34$, $p<.001$, $\varepsilon=0.72$.

Table S2a. *Descriptive statistics for encoding blocks of the Learning Task in Learning Experiment 1b.*

Experiment	Parameter	Block	Test Order	N	Mean	Standard error of the mean
Experiment1b	Accuracy (%)	block1	first	90	98.10	0.20
			second	90	99.20	0.10
		block2	first	90	98.80	0.20
			second	90	99.40	0.10
		block4	first	90	98.60	0.20
			second	90	99.40	0.10
		block5	first	90	98.90	0.20
			second	90	99.60	0.10
	RT (ms)	block1	first	90	910.72	20.79
			second	90	529.18	23.29
		block2	first	90	893.07	19.54
			second	90	474.09	21.18
		block4	first	90	888.96	18.91
			second	90	461.13	21.90
		block5	first	90	882.16	19.98
			second	90	437.49	21.89

Table S2b. *Descriptive statistics for the testing block of the Learning Task in Learning Experiment 1b.*

Experiment	Parameter	Block	Test Order	N	Mean	Standard error of the mean
Experiment1b	Accuracy (%)	block3	first	90	80.40	1.30
			second	90	87.50	1.00
	RT (ms)	block3	first	90	1196.81	22.32
			second	90	814.14	22.18

Table S2c. Repeated Measures Analyses of Variance (rm-ANOVAs) on accuracies and response times from the encoding blocks of the Learning task in Experiment 1b. Significant effects are underlined.

Effects	df1	df2	F	*p*	η^2_p & 95% CI	BF_{10}	interpretation
**Experiment 1b - accuracy**							
Block	2.41	214.56	5.15^{corr}	$.004^{corr4}$	0.05 [0.01, 0.12]	12.20	substantial evidence for the main effect of block compared to a null model
Test order	1	89	65.87	< .001	0.42 [0.27, 0.55]	$2.40\times 10^{+9}$	very strong evidence for the main effect of test order compared to a null model
Block*Test Order	3	267	2.45	0.06	0.02 [0, 0.07]	1.2	Inconclusive evidence
**Experiment 1b – response times**							
Block	1.79	159.33	24.89^{corr}	$<.001^{corr5}$	0.21 [0.11, 0.32]	$2.70\times 10^{+11}$	very strong evidence for the main effect of block compared to a null model
Test order	1	89	1540.89	< .001	0.94 [0.93, 0.96]	$4.26\times 10^{+54}$	very strong evidence for the main effect of test order compared to a null model
Block*Test Order	2.19	195.42	17.11^{corr}	$<.001^{corr6}$	0.16 [0.07, 0.25]	$9.04\times 10^{+7}$	very strong evidence that models with an interaction effect provide a better explanation

Table S2d. One-sample t-test against chance (0.25) on accuracy – test block of the Learning task in Experiment 1b.

	t	df	*p*	Cohen's *d*
**Experiment 1b**				
First object location test	44.14	89	< .001	4.65 [3.93, 5.35]
Second object location test	62.50	89	< .001	6.58 [5.58, 7.55]

[4] Since the sphericity assumption was violated, Greenhouse-Geisser corrected results are reported: $\chi^2(5)=30.62$, $p<.001$, $\varepsilon=0.80$.
[5] Since the sphericity assumption was violated, Greenhouse-Geisser corrected results are reported: $\chi^2(5)=86.45$, $p<.001$, $\varepsilon=0.59$.
[6] Since the sphericity assumption was violated, Greenhouse-Geisser corrected results are reported: $\chi^2(5)=44.81$, $p<.001$, $\varepsilon=0.73$.

Table S3a. *Descriptive statistics for encoding blocks of the Learning Task in Learning Experiment 2.*

Experiment	Parameter	Block	Test Order	N	Mean	Standard error of the mean
Experiment 2	Accuracy (%)	block1	first	85	98.00	0.30
			second	85	99.10	0.20
		block2	first	85	98.00	0.40
			second	85	99.30	0.10
		block4	first	85	97.60	0.30
			second	85	98.90	0.20
		block5	first	85	98.00	0.40
			second	85	99.10	0.20
	RT (ms)	block1	first	85	918.12	24.30
			second	85	501.25	23.05
		block2	first	85	893.00	22.96
			second	85	466.57	24.05
		block4	first	85	897.16	22.94
			second	85	453.95	23.48
		block5	first	85	888.90	23.18
			second	85	433.25	23.28

Table S3b. *Descriptive statistics for the testing block of the Learning Task in Learning Experiment 2.*

Experiment	Parameter	Block	Test Order	N	Mean	Standard error of the mean
Experiment 2	accuracy (%)	block3	first	85	73.10	1.60
			second	85	81.90	1.30
	RT (ms)	block3	first	85	1238.71	26.58
			second	85	848.58	26.46

Table S3c. Repeated Measures Analyses of Variance (rm-ANOVAs) on accuracies and response times from the Learning task. Significant effects are underlined.

Effects	df1	df2	F	*p*	η^2_p & 95% CI	BF_{10}	interpretation
**Experiment 2 - accuracy**							
Block	2.42	203.42	1.50[corr]	0.22[corr7]	0.01 [0, 0.06]	0.16	substantial evidence for the null model
Test order	1	84	36.99	< .001	0.30 [0.15, 0.45]	395716.47	very strong evidence for the main effect of test order comparted to a null model
Block*Test Order	2.69	225.91	0.72[corr]	0.52[corr8]	0.009 [0, 0.04]	0.01	strong evidence for the null model
**Experiment 2 – response times**							
Block	1.98	166.31	16.98[corr]	< .001[corr9]	0.16 [0.07, 0.27]	$2.580\times 10^{+7}$	very strong evidence for the main effect of block compared to a null model
Test order	1	84	1396.26	< .001	0.94 [0.92, 0.96]	$4.892\times 10^{+50}$	very strong evidence for the main effect of test order comparted to a null model
Block*Test Order	2.48	208.43	9.45[corr]	< .001[corr10]	0.10 [0.03, 0.18]	7904.05	very strong evidence that models with an interaction effect provide a better explanation

Table S3d. One-sample t-test against chance (0.25) on accuracy – test block of the Learning task in Experiment 2.

	t	df	*p*	Cohen's *d* & 95% CI
**Experiment 2**				
First object location test	30.30	84	< .001	3.28 [2.74, 3.82]
Second object location test	43.45	84	< .001	4.71 [3.96, 5.44]

[7] Since the sphericity assumption was violated, Greenhouse-Geisser corrected results are reported: $\chi^2(5)=39.04$, p <.001, ε=0.80.
[8] Since the sphericity assumption was violated, Greenhouse-Geisser corrected results are reported: $\chi^2(5)=15.63$, p=.008, ε=0.89.
[9] Since the sphericity assumption was violated, Greenhouse-Geisser corrected results are reported: $\chi^2(5)=64.62$, p <.001, ε=0.66.
[10] Since the sphericity assumption was violated, Greenhouse-Geisser corrected results are reported: $\chi^2(5)=27.72$, p<.001, ε=0.82.
